# Effects of Non-physician Practitioners on Emergency Medicine Physician Resident Education

**DOI:** 10.5811/westjem.58759

**Published:** 2023-05-03

**Authors:** Andrew W. Phillips, Jeremy P. Sites, Faith C. Quenzer, Daniel M Lercher

**Affiliations:** * DHR Health Research Institute, Edinburg, Texas; ‡ University of Kentucky, Department of Pediatrics, Pediatric Critical Care, Lexington, Kentucky; § University of North Carolina at Chapel Hill, Division of Pediatric Critical Care Medicine, Chapel Hill, North Carolina; || San Diego State University, School of Public Health, Division of Epidemiology and Biostatistics, San Diego, California

## Abstract

**Introduction:**

The effects of non-physician practitioners (NPP) such as physician assistants and nurse practitioners on the education of emergency medicine (EM) residents have not previously been specifically evaluated. Emergency medicine societies have made policy statements regarding NPP presence in EM residencies without the benefit of empiric studies.

**Methods:**

A cross-sectional, mixed methods questionnaire with strong validity evidence was distributed to current EM residents who were members of a large national society, the American Academy of Emergency Medicine Resident and Student Association (AAEM/RSA), between June 4–July 5, 2021.

**Results:**

We received 393 partial and complete responses, representing a 34% response rate. A majority of respondents (66.9%) reported that NPPs have a detracting or greatly detracting impact on their education overall. The workload in the emergency department was reported generally as lighter (45.2%) to no impact (40.1%), which was cited in narrative responses as an aspect of both enhancing and detracting from resident physician education. Non-physician practitioner postgraduate programs in EM were associated with a 14x increase in the median number of procedures forfeited over the course of the prior year (median = 7.0 vs 0.5, P<.001). Among respondents, 33.5% reported feeling “not confident at all” in their ability to report concerns about NPPs to local leadership without retribution, and 65.2% reported feeling “not confident at all” regarding confidence in the Accreditation Council for Graduate Medical Education to satisfactorily address concerns about NPPs raised in the end-of-year survey.

**Conclusion:**

Resident members of the AAEM/RSA reported having concerns about the effects of NPPs on their education and their confidence in being able to address the concerns.

## INTRODUCTION

The emergency physician trainee educational environment of both emergency department (ED) and off-service rotations has changed over the last decade with a rapid increase in staffing by non-physician practitioners (NPP), often nurse practitioners and physician assistants.^1^–^3^ Recently the American Academy of Emergency Medicine Resident and Student Association (AAEM/RSA), as well as several other EM and EM resident societies, published policy statements detailing concerns and best practices for the presence of NPPs—and their postgraduate training programs—in EDs with emergency medicine (EM) residents.^4^,^5^ However, there is scant literature assessing the effects of NPPs on physician resident education across the breadth of medicine and no literature specific to EM.

Most prior studies evaluating the impact of NPPs on physician trainee education are from surgical specialties, which consistently report reduced workload, primarily due to reduced documentation responsibilities. Findings are mixed with respect to the impact on residents’ education, with conflicting reports of better operative experiences because of fewer floor pages vs forfeiting some operative procedures to NPs and PAs.^6^–^8^

One survey across an entire academic institution found that NPs reported contributing positively to the education experience of resident physicians.^9^ Another study found a generally positive impact on intensive care unit fellow education according to fellowship directors.^10^ Notably, the residents and fellows whose education was being assessed were not included in either study.

Additionally, an important development across US academic medical centers that has not been captured in any prior studies of resident education to date in any specialty is the increasing number of institutions that now host postgraduate training programs for NPPs.^4^ Early studies have started to evaluate such programs in EM but once again without the perspectives of physician residents, leaving a primary stakeholder unaddressed.^11^,^12^ Our primary objective in this study was to establish how EM residents perceive the effects of NPPs on their education, both while on service in the ED and off service. Our secondary objective was to establish whether those perceptions are associated with the presence vs absence of NPP postgraduate training programs in EM.

## METHODS

In-depth paradata are available in Appendix A. Best practices for survey research were followed and reported using recommended reporting guidelines.^13^,^14^ The study was confirmed to be exempt by the Washington Hospital Health System Institutional Review board.

### Participants and Eligibility

The sampling frame was EM residents at US (state and territory) Accreditation Council for Graduate Medical Education (ACGME)-accredited programs who are members of the American Academy of Emergency Medicine Resident and Student Association (AAEM-RSA), drawn from its files. Residents in combined programs, such as EM/internal medicine, were included as well. Personal leave was not an exclusion criterion since items assessed perspectives over varying amounts of time.

### Survey Method and Validity Evidence

Our primary objective was to capture the physician residents’ perspectives of the effects of NPPs on their education, which is intangible and, thus, best suited for a survey approach.^15^ The instrument was built using established best practices including expert and stakeholder involvement, layout recommendations, cognitive interviews, pilot testing, and nonresponse bias analysis.^16^,^17^ Appendix A contains all relevant paradata.

**Population Health Research Capsule**

What do we already know about this issue?

*Non-physician practitioners (NPPs) are a growing part of the workforce in emergency departments with emergency medicine (EM) residency programs*.

What was the research question?

*What is the impact of NPPs on resident education from the EM resident’s perspective?*

What was the major finding of the study?

*66.9% of residents reported a detracting impact on their education. Presence of an NPP postgraduate program was associated with 14x increase in resident-forfeited procedures*.

How does this improve population health?

*Excellent physician education is critical to addressing population health needs throughout the country*.

We applied Messick’s validity framework consistent with recommendations by the American Association of Public Opinion Research.^18^ Appendix A includes a full validity evaluation, and Appendix B is the final instrument. Educational items were drawn from an existing instrument with good validity evidence,^8^ while gender and race/ethnicity demographics items were drawn from publicly available Association of American Medical Colleges records.^19^ The procedure list was drawn from the ACGME requirements,^20^ and designations of unsupervised NPP practice laws were drawn from a published third-party review study.^21^ A complete list of existing instruments that we considered for use, along with our rationale for inclusion or exclusion in this study, is available in Appendix A.

The survey was open from June 4–July 5, 2021 and distributed electronically via Qualtrics (Qualtrics International, Inc., Provo, UT) with four reminders and an electronic pre-notification the week before the initial invitation, all consistent with best practices.^17^ Although the cohort was AAEM/RSA resident physicians, neither AAEM/RSA as an organization, nor its employees or representatives, were involved in any part of this study including instrument creation and analysis, other than simply distributing the instrument to its members.

### Statistical Analysis

We used skewness and kurtosis to assess whether items met normal distribution requirements for parametric analyses.^22^ The Mann-Whitney U test, chi-square, and Fisher exact tests were used to compare the frequencies of events and scale ratings against the presence of an NPP postgraduate training program, postgraduate year status, and the existence of state laws regarding NPP supervision. We used the Spearman correlation coefficient to assess correlations.

Per published recommendations, we determined a priori to assess for nonresponse bias with both a wave analysis and demographics comparison (Appendix A).^17^ Qualitative analysis was conducted using a holistic coding approach.^23^ Two authors simultaneously developed codes and applied them accordingly (Appendix A). We followed reporting guidelines from *Academic Medicine* for surveys and qualitative data.^13^,^24^

## RESULTS

### Respondents

We received 393 partial and complete responses of 1,168 invitations that were confirmed received and viewed, yielding a 34% response rate. Table 1 shows the respondents’ demographics. State representation is in Appendix C. No empirical evidence for nonresponse bias using two independent analyses was found (Appendix A).

### General Education and Work Experience

Detailed responses to Likert-type items are in Table 2; histograms are shown in Appendix D. Residents reported a generally unchanged or lighter workload and generally unaffected documentation time due to NPPs in the ED. In contrast, more than two-thirds of residents reported a negative impact of NPPs in the ED on their education. Residents reported having limited confidence in the local and national institutions responsible for ensuring the quality of residents’ medical education with respect to the presence of NPPs in the ED. One-third of residents reported feeling no confidence at all in being able to report concerns about the presence of NPPs in the ED without retribution.

### Enhancing and Detracting Educational Impact of Non-physician Practitioners

Responses to the two narrative items evaluating how NPPs in the ED enhance and detract from EM resident education are characterized in Table 3.

### Procedure Experiences in the Emergency Department and Off Service

Appendix E shows the complete numeric breakdown of procedure types and the number of each of those procedures forfeited in the ED and off-service, in addition to histograms for the same information. Table 4 describes the reasons why procedures were forfeited. All narrative responses and their final codes are in Appendix F.

Across all procedures, 264 residents (57.2%) reported at least one procedure for their patient being performed by an NPP during an ED rotation and 220 (59.5%) during an off-service rotation. The median number of procedures being performed by an NPP on residents’ patients in the ED was 2.00, while the off-service median was 2.5. The total number of forfeited procedures correlated inversely with the perception of overall impact on education (ie, forfeited procedures were associated with the perception of detraction from education), r_s_=.381, *P*<.001, r^2^=0.14. Almost one-third (30.5%) of 269 responding residents reported at least one instance during an EM rotation of a patient being preferentially assigned to an NPP. Slightly fewer (25% of 200) reported at least one such instance while off service.

Conversely, 53.3% of 246 respondents reported having at least one patient preferentially assigned to them (physician resident) in lieu of an NPP because of the educational value during an EM rotation. Only 26.1% of 203 respondents reported the same on an off-service rotation. Additionally, of 280 residents who responded to the survey, 15% reported that an NPP taught or supervised them for at least one procedure in the ED, whereas 213 (38%) reported at least one such occurrence while on an off-service rotation.

When asked why procedures were forfeited, unit culture was independently cited significantly more frequently for off-service rotations than for ED rotations (28.0% vs 19.7%, *χ*^2^ (1, n=132)=10.696, *P*<.01, Cramer’s V=0.285). “Intimidation” as a theme was expressed only in the ED responses.

### Presence of EM Postgraduate Training Programs for Non-physician Practitioners

The presence of a postgraduate training program for NPPs was not significantly associated with residents’ impression of the overall impact of NPPs on their education (*P*=.26, Fisher exact test) or on their confidence in being able to report concerns about NPPs to local leadership without retribution (*χ*^2^ (4, n=347)=1.290, *P*=.87). However, EM residents were significantly more likely to have forfeited at least one procedure on their patients than those without such programs on both EM rotations (69% vs 50%, *χ*^2^ (1, n=264)=9.160, *P*<.01, Cramer’s V=.186) and off-service rotations (68.8% vs 54.3%, *χ*^2^ (1, n=220)=4.422, *P*=.04, Cramer’s V=.142). Significantly more total procedures in the ED were forfeited as well by residents whose institutions hosted an NPP postgraduate EM program (median = 7.0) compared to not [median =0.5, *U(N_with_*=100, *N_without_*=164)=6,070.5, z=−3.687, P<.001, η^2^=.052], a factor of 14x, and trended similarly for reports of off-service procedures [median =7.5 with vs 2.0 without, *U(N_with_*=80, *N_without_*=140)=4,769.0, z=−1.894, *P*=.06].

Twice as many residents reported forfeiting at least one educational ED patient encounter at programs with an associated NPP postgraduate program than those without (43.6% vs 21.3%, *χ*^2^ (1, n=249)=13.965, *P*<.001, Cramer’s V=0.237), but the reverse was not true for patients being preferentially assigned to residents (59.3% vs 47.6%, *χ*^2^ (1, n=229)=2.970, *P*=.09). The presence of an NPP postgraduate EM program was not significantly associated with the incidence of teaching or supervision by an NPP in the ED (18.8% vs 11.7%, with and without, respectively, *χ*^2^ (1, n=259)=2.483, *P*=.12).

### EM Resident Postgraduate Training Status

Postgraduate year (PGY) status was not associated with a difference in probability of having forfeited a procedure to an NPP in the ED but was during off-service rotations, with 63.0%, 70.9%, and 47.1% of PGY 1, 2, and 3+, respectively, reporting at least one forfeiture, *χ*^2^ (2, n=237)=10.101, *P*<.01, Cramer’s V=0.206. There was no significant difference in the incidence of forfeiting at least one highly educational patient to NPPs across PGY status in the ED [30.0%, 29.1%, 30.4% for PGY 1, 2, 3+, respectively, *χ*^2^ (2, n=251)=0.036, *P*=.98] or off-service rotations [20.6%, 9.5%, 9.0% for PGY 1, 2, 3+, respectively, *χ*^2^ (2, n=199)=1.081, *P*=.58].

## DISCUSSION

A substantial majority of EM residents in AAEM-RSA reported that NPPs in the ED have a detracting or greatly detracting impact on their education. The presence of an EM NPP postgraduate training program was associated with a significantly greater median number of forfeited procedures but not with effects of NPPs on resident perception of education. Additionally, more than one-third of residents reported feeling “not confident at all” that they could approach local leadership about NPP concerns without facing retribution, and almost two-thirds of residents were “not confident at all” that the ACGME would satisfactorily address concerns about NPPs impacting resident education as reported in the oversight body’s year-end survey.

Of further interest are the measures that were not statistically significant. For example, the data do not show that more experienced senior residents are the ones primarily forfeiting procedures; rather, there was no significant difference by PGY status. Additionally, a higher number of forfeited procedures was significantly associated with a negative effect of NPPs on education, and NPP postgraduate programs were significantly associated with a higher number of forfeited procedures. Nonetheless, NPP postgraduate programs were not significantly associated with a difference in the overall perception of NPP effects on resident education. This finding suggests there is at least one mitigating factor of NPP postgraduate programs that balances the loss of procedures.

Of note, forfeited procedures reported here are for those patients the residents were primarily managing, procedures for whom the residents were ostensibly responsible. The number of such forfeited procedures was moderately associated with residents’ perception of educational effects, accounting for 14% of the variance; nevertheless, the confluence of data suggests a phenomenon that is far more complex than frustration over fewer opportunities for procedures.

The narrative responses were telling with respect to the hidden curriculum, which is generally described as a construct for the effects of tacit learning as a confluence of culture, structures, and institutions.^25^ Intimidation and unit-culture themes suggest a new facet for a hostile learning environment and conditions that appear to leave physician trainees feeling defenseless. The example quotation from Table 4—“They just push their way in and tend to have the support of the administration”—points to a structured, even if unintentional, hidden curriculum that is a hindrance to physician resident education.

The narrative responses also described a loop of exclusion in which residents were told at times that they were required to forfeit their procedure so an NPP could have more experience but also reported times in which they were required to forfeit their procedure because the NPP had more experience than the resident. Findings from the Kang et al study alluded to a similar phenomenon in the operating room for junior residents,^8^ and it is thus not surprising that narrative responses described problematic relationships with NPPs on off-service rotations as well, putting at jeopardy the value-add of off-service rotations for EM residents.

Comparison to the study of surgical residents by Kang and colleagues bears striking contrasts across items that were replicated in our instrument.^8^ A full 88% of their respondents reported that NPPs made their workload lighter or much lighter, compared to 46.5% in our study. Similarly, 86% of surgical residents reported that NPPs enhanced or greatly enhanced care, whereas only 18.5% of emergency physicians shared the same opinion in the ED setting. Finally, 47% of surgical residents felt that NPPs enhanced or greatly enhanced their education (with 47% reporting no impact), whereas 66.9% of EM residents reported that an NPP presence detracted or greatly detracted from their education (with 29.8% reporting no impact), which is essentially the inverse of the surgical findings. The perceived workload and educational benefits found in the surgical specialties are not translated in EM from the perspective of EM residents.^6^,^8^ The specialized and largely procedural nature of surgical education is distinct from the breadth of case exposure required for EM education. The contrast makes clear that surgical and EM resident cohorts are different, and conclusions cannot be inferred across the two groups.

The conflicting findings in the surgical literature of reduced workload on the one hand but reduced procedural opportunities on the other was present in EM residents’ responses as well. One of the most frequently cited educational enhancements provided by NPPs in the ED (17.9%) was reduced workload via fewer lower acuity patients to see, thereby allowing an educational emphasis for residents on more complex patients. By the same token, however, one of the most frequently cited detractions from education as a result of NPPs in the ED (47.7%) was the reduction of cases, including lower acuity cases.

As EM societies grapple with this issue, identifying institutional features of the reported positive interactions will be essential to inform best practices to improve the team relationship.^4^ Within that context, two aspects of structured interaction between NPPs and EM residents must be independently addressed: 1) NPPs in the ED as staff; and 2) NPPs as postgraduate trainees. One resident response in particular was telling with regard to the potential negative impact of the postgraduate programs on physician residents, given the recent report of a novel, parallel track postgraduate physician assistant program:^11^ “They are in a parallel training environment with different standards and often give [physician residents] sub-par advice or worse, aggressive sub-par advice because they consider themselves more advanced.” It is likely sentiments such as these from physician residents that have led to the AAEM-RSA calling for the cessation of NPP postgraduate programs.^26^

## LIMITATIONS

Our survey asked the survey participants for recall over the course of a full year, which raises the potential for recall bias; however, none was found on the pilot test/retest analysis, supporting item reliability. An additional limitation is that our sampling frame was of a group that did not include every resident in the US and whose members are part of a specific EM society. It is notable that the sampling frame still represents almost three-fifths of all EM residents in the US, a large group indeed. Additionally, most AAEM-RSA members have membership through their programs, suggestive of those programs supporting less involvement of NPPs in resident education if consistent with AAEM and AAEM-RSA position statements. Thus, our findings would be underestimates of the detracting educational effects of NPPs on resident education and of forfeited procedures. It is also worth noting that the other major resident societies, the Emergency Medicine Residents’ Association and the American College of Osteopathic Emergency Physicians’ Resident Student Organization, also signed on to the letter regarding NPP involvement in resident education, underscoring that there is clearly not a bias of our particular cohort.^4^

Importantly, in this study we evaluated how many procedures and patient opportunities were *lost* but did not count how many were experienced in total, which is an undoubtedly larger and similarly consequential number. Fourteen lost procedures in a year could represent any percentage; the denominator is unknown. Finally, our study focused on the educational aspect of NPPs in the ED from the perspective of EM residents. Staffing models must also account for throughput, cost, and myriad other factors.

### Future Study

Although our study focused on physician residents because they were not previously studied, all stakeholders—including physician residents, attendings, staff NPPs and NPPs in postgraduate programs, medical directors, and department administrators—must be included in addressing what residents report to be a hindrance in their education. Additional study and intervention are warranted regarding residents’ lack of confidence in local leadership and the ACGME. Finally, the findings in this study provide tangible evidence of the theoretical concerns raised by the major EM societies.

## CONCLUSION

A strong majority of resident members of AAEM-RSA report that non-physician practitioners in the ED have a detracting impact on their overall education and opportunities for learning cases and procedures, at least in part because of preferential treatment of NPPs. Educational enhancement was reported but limited. Residents overwhelmingly do not have confidence in local or national authorities to address potential concerns about NPPs in the ED impacting their education.

## Supplementary Information













## Figures and Tables

**Table 1:** Demographic features of survey respondents.

Category	No. (%)
Postgraduate year	
1	94 (32.1%)
2	89 (30.4%)
3	75 (25.6%)
4	32 (10.9%)
5	3 (1.0%)
Gender	
Male	187 (65.4%)
Female	99 (34.6%)
Race/ethnicity	
Asian	31 (11.0%)
Black	12 (4.3%)
Hispanic, Latino, other, Pacific Islander	12 (7.4%)
White	195 (69.1%)
Other race/ethnicity	19 (6.7%)
Unknown race/ethnicity	3 (1.1%)
Non-US citizen or non-permanent resident	1 (0.4%)
State NPP supervision laws	
Independent	80 (29.7)
Supervised	189 (70.3)
Post-Graduate program for NPs and/or PAs	
Yes	133 (22.9)
No	214 (36.8)
Don’t know	32 (5.5)

* Total n for each item varies due to item nonresponse.

*NPP*, non-physician practitioner; *PA*, physician assistant.

**Table 2:** Likert-type responses to items assessing the impact of non-physician practitioners in the emergency department on general education and work experience.

Effect on resident workload in the emergency department (n=392)				
Much lighter	Lighter	No impact	Heavier	Much heavier
1.3	45.2	40.1	12.2	1.3

Effect on resident documentation time (n=392)				

Greatly decrease	Decrease	No effect	Increase	Greatly increase
1.0	4.8	80.6	11.5	2.0

Effect on patient care in the emergency department (n=391)				

Greatly detract	Detract	No impact	Enhance	Greatly enhance
11.8	45.0	24.8	18.2	0.3

Effect on resident education (n=393)				

Greatly detract	Detract	No impact	Enhance	Greatly enhance
19.3	47.6	29.8	3.1	0.3

Confidence in ability to report concerns about NP/PA presence to local leadership without retribution (n=379)				

Not confident at all	A little confident	Moderately confident	Quite confident	Extremely confident
33.5	26.4	17.9	15.8	6.3

Confidence in the ACGME to satisfactorily address concerns about NP/PA presence reported in the annual end-of-year survey (n=379)				

Not confident at all	A little confident	Moderately confident	Quite confident	Extremely confident
65.2	24.8	7.1	2.1	0.8

* Percentage values are reported as a function of the total item responses.

*NP*, nurse practitioner; *PA*, physician assistant.

**Table 3:** Ways in which non-physician practitioners enhance and detract from resident education in the emergency department.

	Theme	No. (%)	Example(s)
Enhance			
	No enhancement	197 (62.9%)	“There is no conceivable way that the presence of NP/PA enhances resident education.”
	Offload lower acuity patients	56 (17.9%)	“[NPPs] frequently run fast track, which opens the opportunity to see sicker patients without being overloaded with lower acuity complaints.”
	Miscellaneous	34 (10.9%)	“We work very independently from the PAs/NP in our department. They cover the ED during resident conference days, so in that way they allow us time for education. However, at our particular institution they do not move patients through the department at quite the same speed as the physicians and so often we come onto shift after conference to a very busy board.”
	Resource/Experienced for advice	24 (7.7%)	“They have knowledge of the system when you’re starting out.” “Some PAs have previous experience of working other specialties and can provide clinical insight as well as tips/tricks.”
	Practice overseeing NPP	14 (4.5%)	“Enhances my sense of the dynamic between attending practitioners and APPs, something I am sure I will deal with later in my career.” “....practice leading APP practitioners before graduation.”
Detract			
	Fewer patient encounters for learning	155 (47.7%)	“[NPPs] take all the procedures without seeing the patients.”
	Fewer procedural opportunities	122 (37.5%)	“None.”
	No detraction	72 (22.2%)	“None.”
	Miscellaneous	36 (11.1%)	“I’m expected to spend time educating NP/PA students to train my replacements.” “I end up teaching them. I taught one how to do a pelvic exam!”
	Monopolizing attending time	31 (9.5%)	“APPs in the ED take up time and energy from Attending Physician [sic] who need to supervise them. This is time that could be directed at resident education and supervision.”
	Hostile learning environment	29 (8.9%)	“Talk down to residents…” “Aggressively lobbying leadership for autonomy.” “They are in a parallel training environment with different standards and often give sub-par advice or worse, aggressive sub-par advice because they consider themselves more advanced.”

*NPP*, non-physician practitioner; *APP*, advanced practice practitioner; *PA*, physician assistant; *NP*, nurse practitioner.

**Table 4:** Reasons procedures were forfeited by emergency medicine residents to non-physician practitioners in the emergency department and while off-service.

	Theme	No. (%)	Example(s)
Emergency department			
	NPP does not offer	20 (10.2%)	“Off-service APPs generally from the trauma service covering during surgical conferences generally will not defer to ED residents for procedures during trauma resuscitations”
	Intimidation	15 (7.6%)	“They just push their way in and tend to have the support of the administration.” “PA/NP insisted that it was their procedure, and I did not think I was in a position to speak back to them.”
	Direct competition/trainee	61 (31%)	“For the PA ‘fellow’ to get more experience.” “The NP/PA asked the attending to do the procedure as part of their training, but they could not take the patient as a primary because of their current volume load.”
	Unit culture	32 (16.7%)	“Customary at that institution. I was a rotator.” “Some attendings preferred to work with non-physician [practitioners] who they had more experience with than a resident who they only knew for a short period.”
	Miscellaneous	18 (9.1%)	“Time.” “Because the attending was busy and couldn’t supervise.”
	None	63 (32%)	“None.”
Off-service			
	Attending comfort with NPP	14 (7.9%)	“ICU, NP/PA had priority due to attending comfort with them.”
	NPP more experienced	14 (7.9%)	“The PA/NP was more experienced.” “More training.”
	Direct competition/trainee	29 (16.4%)	“CRNA took anesthesia intubations and only let CRNA students intubate over EM residents.” “For their educational value.”
	NPP stole procedure	7 (4.0%)	“There was no reason-they stole it.”
	Unit culture	52 (29.7%)	“They worked on the unit and oversaw procedures.” “My senior resident in the MICU was not credentialed to do central lines, fellow/attending were not in house overnight. NPs are not technically allowed to supervise us so she put the central lines in overnight.”
	Miscellaneous	28 (15.0%)	“Division of labor. I was doing other stuff.” “I was staffing the PA/NP.”
	No reason	51 (29.0%)	“None” “No reasons”

*NPP*, non-physician practitioner; *APP*, advanced practice practitioner; *PA*, physician assistant; *NP*, nurse practitioner; *ICU*, intensive care unit; *CRNA*, certified registered nurse anesthetist.
