# Supplementary material for: Effects of Non-physician Practitioners on Emergency Medicine Physician Resident Education
Source: West J Emerg Med. 2023 May 3;24(3):588–96. doi: 10.5811/westjem.58759 (PMC10284528; doi:10.5811/westjem.58759)
Supplement: Supplementary file 1 [file wjem-24-588-s001.docx]

APPENDIX A. PARADATA

*Sampling Frame Decisions*

The study population of interest is EM residents in the United States. The American Academy of Emergency Medicine Resident and Student Association (AAEM/RSA) is a national organization of emergency physician residents across the US. Although not all residents in the US are members of the organization, nonresponse bias analyses were performed against all of emergency medicine (the ACGME data) so as to evaluate for both nonresponse bias and sampling bias.

The Emergency Medicine Residents Association (EMRA) is the largest EM resident organization and was first approached for distribution as the sampling frame but was unable to meet instrument delivery requirements.

*Summary of Prior Instruments Reviewed for Inclusion*

Existing instruments:

| 1^st^ Author | Cohort | Setting | Usability |
| --- | --- | --- | --- |
| Joffe | Crit care PDs (not trainees) | ICU | No—wrong cohort |
| Bahouth | NPs, nurses/residents (combined) | Whole center | No—wrong cohort |
| Fang | Residents single center | IM (surgical comanagement service) single center | No—evaluated impact of faculty vs NPP on workload |
| Kahn | Surgical residents | ICU ?nationally? does not specify distribution | No—atypical scales (0-100) without reported reliability; items described appeared biased toward NPP favorability (e.g., asked positive effects but did not ask negative effects) |
| Kang | Surgical residents | Single center | Yes, instrument created described and validated |
| Holleman | Attendings, PICU fellows (2/25), nurses, charge nurses | Single center, single NP on service | no—wrong cohort |
| Eaton | Surgical residents and fellows, NPPs | Surgical service line single center | Potentially for specific items; domains not clear |

We attempted to contact the authors of the Kang et al. and Eaton et al. studies to obtain original survey instruments, but e-mail addresses for all authors were either incorrect or no response was received. Phone messages were left for each author as well between November 30 and December 1, 2020, although this method of contact was especially hindered by remote work during the COVID-19 pandemic. However, some of the items in the Kang et al. study were written verbatim in the manuscript; these selected items were used verbatim in our final instrument.

*Survey Development*

The instrument began with the selected items from Kang and creation of items based on previously published resident procedure requirements. Specifically, taken verbatim including response options except as in brackets in which words were added were the following items:

- “Overall, how does the presence of NP/PAs affect your workload [in the emergency department]?”
- “How do NP/PAs affect your time spent on documentation?”
- “How do you think NP/PAs affect patient care [in the emergency department]?”
- “Overall, what impact do NP/PAs have on your education?”

Additional items were created based on the literature search and authors’ knowledge of emergency medicine practice. The primary author of the instrument (AWP) is experienced in survey development and has numerous publications on the topic. The collaborating author of the instrument (FCQ) is an experienced researcher who completed a research fellowship. The initial draft was reviewed by an internationally recognized survey expert using a formal, expert evaluation rubric previously published (Phillips, Durning, and Artino. Survey Methods for Medical and Health Professions Education. Elsevier. 2021). The draft was also reviewed by an assistant program director of emergency medicine. Cognitive interviews with a combined think-aloud and verbal probing approach were conducted by AWP with two 2020 emergency medicine residency graduates with different backgrounds to ensure interpretation of the items. Based on those cognitive interviews, four additional items were added to the instrument, asking how often during ED and off-service rotations patients of particular educational value were preferentially given to residents and how often they were preferentially given to NPPs. Pilot test responses (n=7) by board members of the Young Physician Section of the American Academy of Emergency Medicine produced results consistent with expectations based on item wording, and no concerns were raised by those who completed the pilot test.

*Demographic Comparisons*

The AAMC Report on Residents references the ACGME full report. There was a discrepancy between online tables and those in the complete PDFs regarding sex and gender. Sex was the label but appeared to be actually describing gender in the PDF because a nonbinary option was available. The online table only includes male and female and is labeled “sex.” Several attempts were made to clarify with the AAMC without reply.

*Survey Delivery Specifics*

The AAEM/RSA communication database was filtered for current emergency medicine residents in US-based ACGME-accredited residency programs. A pre-notification was sent to the sampling frame by the AAEM/RSA president the week before the initial launch. The first invitation was sent 6/4/21, followed by reminders 6/7/21, 6/13/21, 6/17/21, and 6/28/21. The survey opened on 6/4/21 and closed 7/5/21, extended into July to capture the holiday weekend for the last wave of respondents.

*Overall Data Handling Rules and Decisions*

Any range provided for a numerical item was entered as the higher number to capture the full possible range. “None” and “never” were coded as 0. “Few” and “rarely” were coded as 3. “Several” was coded as 5. “Numerous,” “many,” and “lots of” were coded as 10. “Countless” was coded as 99, the maximal amount. Responses of “?” and “I don’t know” were coded as missing data. One case reported “new resident” to items asking about the past year’s experience. The entire case was removed since it did not appear that the resident had sufficient EM resident experience to respond to the items. No items required the removal of more than 5 responses for uninterpretable qualifiers.

*Data Handling for Specific Items*

Item 15 (Since July 2020, how many times on an emergency medicine rotation have each of the following procedures been performed on *your* patient by an NP or PA when you were available to do the procedure?) was a narrative response with requested numbers but without a numeric lock because of platform limitations. One case used text descriptions, such as “most,” and those instances were changed to missing data.

Item 12 (Since July 2020, how many times on an emergency medicine rotation has an NP or PA taught or supervised you performing one of the above procedures?) Several respondents added comments such as “I will never allow this to happen to me.” One respondent only wrote “most happen off service” but did not provide a number; this was coded as missing data.

Item 13 (Since July 2020, how many times on an emergency medicine rotation was a patient assigned to an NP or PA instead of you because of the high educational value of taking care of that patient?) also received some text responses. Three cases reported that patients are not assigned at their institution; the data were coded as missing.

Item 33 (Since July 2020, how many times on an emergency medicine rotation was a patient assigned to you instead of an NP or PA because of the high educational value of taking care of that patient?) is unlikely an accurate accounting of the event. The responses implied a variety of interpretations ranging from initial triage of patients to ESI levels which are then seen by physicians or non-physicians to a more granular interpretation to specific cases. One reply was “this is the norm at my program,” but had to be coded as missing data since a number was not provided. Another respondent replied “80%” which had to be coded as missing data as well since no absolute number could be applied to the analysis. Any data such as the above examples that did not suggest an absolute number was coded as missing. Two cases reported that patients are not assigned; the data were coded as missing. This was the only item for which “countless” was the response.

Item 35 (Since July 2020, how many times on an off-service rotation have each of the following procedures been performed on *your* patient by an NP or PA when you were available to do the procedure? Was treated exactly the same as Item 15 described above. One respondent answered “often” to central venous catheters and chest tubes, which had to be coded as missing data since no absolute number can be inferred. One respondent replied “70%” to adult medical resuscitations, “85%” to dislocation reduction, “95%” to pediatric medical resuscitation, and “60%” to vaginal deliveries. These data were interpreted as missing since no absolute number could be inferred.

Item 17 (Since July 2020, how many times on an off-service rotation has an NP or PA taught or supervised you performing one of the above procedures?) had one response of a description, “CRNAs on Pediatric Anesthesia. 2 Weeks,” but no additional details so was coded as missing data. A few respondents answered with similar qualifying statements to the corollary item for EM practice, stating, for example, “I will not allow this to happen to me,” and another stated that the supervision was declined. One respondent remarked that there were no off-service rotations with non-physician practitioners.

Item 20 (Since July 2020, how many times on an off-service rotation was a patient assigned to an NP or PA instead of you because of the high educational value of taking care of that patient?) again had a few qualifying remarks such as “often” that required removal, but only 2 such cases. A single case did not provide a frequency or absolute number but described the occurrence in the ICU because the non-physician practitioners were “more ‘experienced.’”

Item 32 (Since July 2020, how many times on an off-service rotation was a patient assigned you instead of an NP or PA because of the high educational value of taking care of that patient?) like Item 33 suffered similarly mixed interpretations.

*Narrative Item Coding*

One author (AWP) taught and oversaw coding by two other authors (DML and JPS) who performed an initial review of responses, then coded simultaneously, occasionally seeking a tie-breaking opinion from AWP. DML and JPS were intentionally selected because they are pediatricians who have practiced in the ED but are not emergency physicians and so less likely to be biased in their coding as a result of commentary about the practice of NPPs in the ED within the EM community. Coding was performed in Microsoft Excel by hand.

*Nonresponse Bias*

Anecdotal reports, cognitive interviews, and our literature search suggested that this topic is controversial with strong opinions of varying perspectives. This plus the notable salience of the topic for residents put the study at relatively high risk of nonresponse bias.

We determined *a priori* to use Q2 “Overall, what impact do NP/PAs have on your education?” as the wave analysis item because it was best-suited to capture strong general favorable vs unfavorable opinions of NPs and PAs in the context of resident education. The first wave was defined as June 7, 2021 (the day the survey opened) through June 12, 2021 (the day before the next reminder was sent); the last wave was defined as June 28, 2021 (the day the last reminder was sent) through July 5, 2021 (the day the survey was closed). No difference was found in the distribution of responses between waves, 𝜒^2^ (3, n=242)=0.962, *p*=.811. Chi-square was used because the frequencies met minimum requirements with only one cell having a count <5.

Demographic information for sex and race/ethnicity, as defined by the AAMC and ACGME were available and used as an opportunity to evaluate for both nonresponse bias and selection bias, suggesting generalizability of the findings to the population of emergency medicine residents. Calculations were made based on percentages. No difference was found between respondents and AAMC data with respect to sex [𝜒^2^ (1)=0.022, *p*=.883] or ethnicity [𝜒^2^ (6)=9.810, *p*=.133].

*Validity Evidence*

The *content evidence* was established in a few ways. First, a literature search of PubMed and Google Scholar produced several prior survey instruments of which one had a domain (“clinical experience”) that addressed our objectives. In addition to items from the “clinical experience” domain used from Kang et al., with minimal change, the demographics items for sex and race/ethnicity were taken from the publically available Association of American Medical Colleges (AAMC) records, and the procedure list taken verbatim from the ACGME key index procedure minimums list (references in manuscript). All other items were borne of literature review, informal interviews, and informal focus groups. The nurse practitioner supervision laws were drawn from a 2019 review because implementation of laws takes time to come into effect (referenced in manuscript). The *response process* evidence was established using cognitive interviews with two 2020 EM residency graduates with different backgrounds that further informed item drafts and by expert reviews by an additional survey expert who is well-published in the field and an assistant program director. Additionally, a pilot test with members of the AAEM Young Physicians Section was conducted to ensure data ranges, user experience, and data output. The pilot test was repeated 1 month after the initial invitation to evaluate test-retest reliability as a guard against recall bias and was found to be acceptable. The consideration of *relationships with other variables* in Messick’s framework was limited by the lack of other studies on the topic in emergency medicine but was evaluated against studies of other specialties. Evidence of the *internal structure* was sufficient based on the correlation between key items; the *consequences* component of the framework is discussed at length in the discussion.

The Kang et al. study did not include all items for its domains so they could not be used for comparison. Additionally, our instrument was not built out into domains, but rather individual concepts evaluated with different measures such as frequency of events along with Likert-type items. The evidence for *internal structure* and *relationships with other variables* was deemed sufficiently strong, however. The two workload-related items (workload and documentation time) were moderately correlated (r_s_=.293, p<.001). The overall impact on education moderately correlated (inversely) with the number of ED procedures residents reported forfeiting (r_s_=-.381, p<.001). Additionally, the total procedures forfeited in the ED correlated moderately well with the total procedures forfeited during off-service rotations r_s_=.410, p<.001).

Test-retest reliability was evaluated for key items using Fisher’s exact test for ordinal variables because there was insufficient variance for a meaningful ICC or Spearman’s rho. The sessions were 6 weeks apart. The workload item was not significantly different (p=1.000, *Fisher’s exact test*); the overall impact on education item was not significantly different (p=.500, *Fisher’s exact test*); the patient care item was not significantly different (p=.167, *Fisher’s exact test*); the documentation time item was not significantly different (p=1.000, *Fisher’s exact test*); the confidence in local leadership item was not significantly different (p=1.000, *Fisher’s exact test*); the confidence in the ACGME item was not significantly different (p=1.000, *Fisher’s exact test*). Missing data from the items asking for a specific number of procedures forfeited precluded testing those items.
