# Supplementary material for: Effects of Non-physician Practitioners on Emergency Medicine Physician Resident Education
Source: West J Emerg Med. 2023 May 3;24(3):588–96. doi: 10.5811/westjem.58759 (PMC10284528; doi:10.5811/westjem.58759)
Supplement: Supplementary file 2 [file wjem-24-588-s002.pdf]

Introduction

The following is an anonymous, voluntary, brief survey evaluating the educational impact of nurse practitioners (NPs) and physician assistants (PAs), also referenced as “advanced practice providers” (APP) and “nonphysician practitioners” (NPP) during your emergency medicine residency.

When responding to the survey items, please include NPs and PAs who were employed (including during “onboarding”), part of a post-graduate training program, or students.

EMERGENCY DEPARTMENT

The following questions pertain to your **emergency department rotations** during **all of your residency thus far unless otherwise specified**. Please do **NOT** include experiences on off-service rotations.

Overall, how does the presence of NP/PAs affect your workload in the emergency department?

- Much lighter
- Lighter
- No impact
- Heavier
- Much heavier

How do NP/PAs affect your time spent on documentation?

- Greatly decrease
- Decrease
- No effect
- Increase
- Greatly increase

How do you think NP/PAs affect patient care in the emergency department?

- Greatly detract
- Detract
- No impact
- Enhance
- Greatly enhance

Overall, what impact do NP/PAs have on your education?

- Greatly detract

- Detract
- No impact
- Enhance
- Greatly enhance

The next two questions ask for more detailed information on the impact of NPs and PAs for EM resident education.

In what ways does the presence of NP/PAs in the emergency department **detract** from your education?

Please write “none” if there is no detraction from your education.

In what ways does the presence of NP/PAs in the emergency department **enhance** your education?

Please write “none” if there is no enhancement to your education.

How confident are you that you can report any concerns, if any, to your local leadership about the presence of NP/PAs in the emergency department without retribution (formal or informal)?

- Not confident at all
- A little confident
- Moderately confident
- Quite confident
- Extremely confident

How confident are you that the ACGME will satisfactorily address any concerns, if any, about the effect of NP/PAs on your education if reported in the annual year-end survey?

- Not at all confident
- Slightly confident
- Moderately confident
- Quite confident
- Extremely confident

Does an emergency department at which you rotate also currently have an emergency medicine post-graduate training program for NPs and/or PAs?

- Yes
- No
- I don't know

Please answer the following questions based rotations in the **emergency department** during this academic year, **starting July 2020**.

Since July 2020, how many times on an **emergency medicine rotation** have each of the following procedures been performed on ***your*** patient by an NP or PA when you were available to do the procedure (0-99 allowed)? Include consultant NPs and PAs (such as in the trauma bay) as well.

	Number of Times<br>Enter 0-99
Adult medical resuscitation	
Adult trauma resuscitation	
Cardiac pacing	
Central venous access	
Chest tubes	
Cricothyrotomy	
Dislocation reduction	
ED bedside ultrasound	
Intubations	

Lumbar puncture	
Pediatric medical resuscitation	
Pediatric trauma resuscitation	
Pericardiocentesis	
Procedural sedation	
Vaginal delivery	

What were the reasons for a procedure being performed on your patient by an NP or PA when you were available to do the procedure?

Since July 2020, how many times on an **emergency medicine rotation** has an NP or PA taught or supervised you performing one of the above procedures? (Enter number 0-99.)

Since July 2020, how many times on an **emergency medicine rotation** was a patient *assigned to an NP or PA instead of you* because of the high educational value of taking care of that patient? (e.g., so the individual could do the necessary procedures) (Enter number 0-99.)

Since July 2020, how many times on an **emergency medicine rotation** was a patient *assigned to you instead of an NP or PA* because of the high educational value of taking care of that patient? (e.g., so you could do the necessary procedures) (Enter number 0-99.)

OFF-SERVICE ROTATIONS

The following questions pertain to your **off-service rotations** during residency. Please do **NOT** include experiences in the emergency department. Please answer each of the following items as they apply to your experience **since July 2020** (this academic year).

Since July 2020, how many times on an **off-service rotation** have each of the following procedures been performed on *your* patient by an NP or PA when you were available to do the procedure (0-99 allowed)?

	Number of Times<br>Enter 0-99
Adult medical resuscitation	
Adult trauma resuscitation	
Cardiac pacing	
Central venous access	

Chest tubes	
Cricothyrotomy	
Dislocation reduction	
ED bedside ultrasound	
Intubations	
Lumbar puncture	
Pediatric medical resuscitation	
Pediatric trauma resuscitation	
Pericardiocentesis	
Procedural sedation	
Vaginal delivery	

What were the reasons for a procedure being performed on your patient by an NP or PA when you were available to do the procedure?

Since July 2020, how many times on an **off-service rotation** has an NP or PA taught or supervised you performing one of the above procedures? (Enter number 0-99.)

Since July 2020, how many times on an **off-service rotation** was a patient *assigned to an NP or PA instead of you* because of the high educational value of taking care of that patient? (e.g., so the individual could do the necessary procedures) (Enter number 0-99.)

Since July 2020, how many times on an **off-service rotation** was a patient *assigned you instead of an NP or PA* because of the high educational value of taking care of that patient? (e.g., so you could do the necessary procedures) (Enter number 0-99.)

Final Section: Demographics

What is your current PGY status?

- PGY1
- PGY2
- PGY3
- PGY4
- PGY5
- PGY6
- PGY7

- PGY8
- PGY9
- PGY10

What is your sex? (Options are drawn from AAMC data.)

- Male
- Female

Which of the following best describes your race/ethnicity? (Options are drawn from AAMC data.)

- American Indian or Alaska Native
- Asian
- Black or African American
- Hispanic, Latino, or Other Pacific Islander
- White
- Other Race/Ethnicity
- Unknown Race/Ethnicity
- Non-US Citizen or Non-Permanent Resident

In which state or territory is your residency program?

Powered by Qualtrics
