# Supplementary material for: Effects of Non-physician Practitioners on Emergency Medicine Physician Resident Education
Source: West J Emerg Med. 2023 May 3;24(3):588–96. doi: 10.5811/westjem.58759 (PMC10284528; doi:10.5811/westjem.58759)
Supplement: Supplementary file 3 [file wjem-24-588-s003.docx]

APPENDIX C. Respondents by Individual States

| **State** | **Frequency** | **Percent** |
| --- | --- | --- |
| Alabama (AL) | 1 | 0.4 |
| Arizona (AZ) | 6 | 2.2 |
| California (CA) | 35 | 13.0 |
| Connecticut (CT) | 7 | 2.6 |
| District of Columbia (DC) | 8 | 3.0 |
| Florida (FL) | 13 | 4.8 |
| Georgia (GA) | 3 | 1.1 |
| Illinois (IL) | 24 | 8.9 |
| Indiana (IN) | 3 | 1.1 |
| Iowa (IA) | 2 | 0.7 |
| Kentucky (KY) | 3 | 1.1 |
| Louisiana (LA) | 2 | 0.7 |
| Maryland (MD) | 8 | 3.0 |
| Massachusetts (MA) | 5 | 1.9 |
| Michigan (MI) | 21 | 7.8 |
| Minnesota (MN) | 7 | 2.6 |
| Mississippi (MS) | 2 | 0.7 |
| Missouri (MO) | 13 | 4.8 |
| New Jersey (NJ) | 6 | 2.2 |
| New York (NY) | 29 | 10.8 |
| North Carolina (NC) | 8 | 3.0 |
| Ohio (OH) | 11 | 4.1 |
| Oklahoma (OK) | 2 | 0.7 |
| Pennsylvania (PA) | 20 | 7.4 |
| Rhode Island (RI) | 6 | 2.2 |
| Tennessee (TN) | 4 | 1.5 |
| Texas (TX) | 9 | 3.3 |
| Virginia (VA) | 3 | 1.1 |
| Washington (WA) | 5 | 1.9 |
| West Virginia (WV) | 1 | 0.4 |
| Wisconsin (WI) | 2 | 0.7 |
