# Supplementary figures and images for: Effects of Non-physician Practitioners on Emergency Medicine Physician Resident Education
Source: West J Emerg Med. 2023 May 3;24(3):588–96. doi: 10.5811/westjem.58759 (PMC10284528; doi:10.5811/westjem.58759)

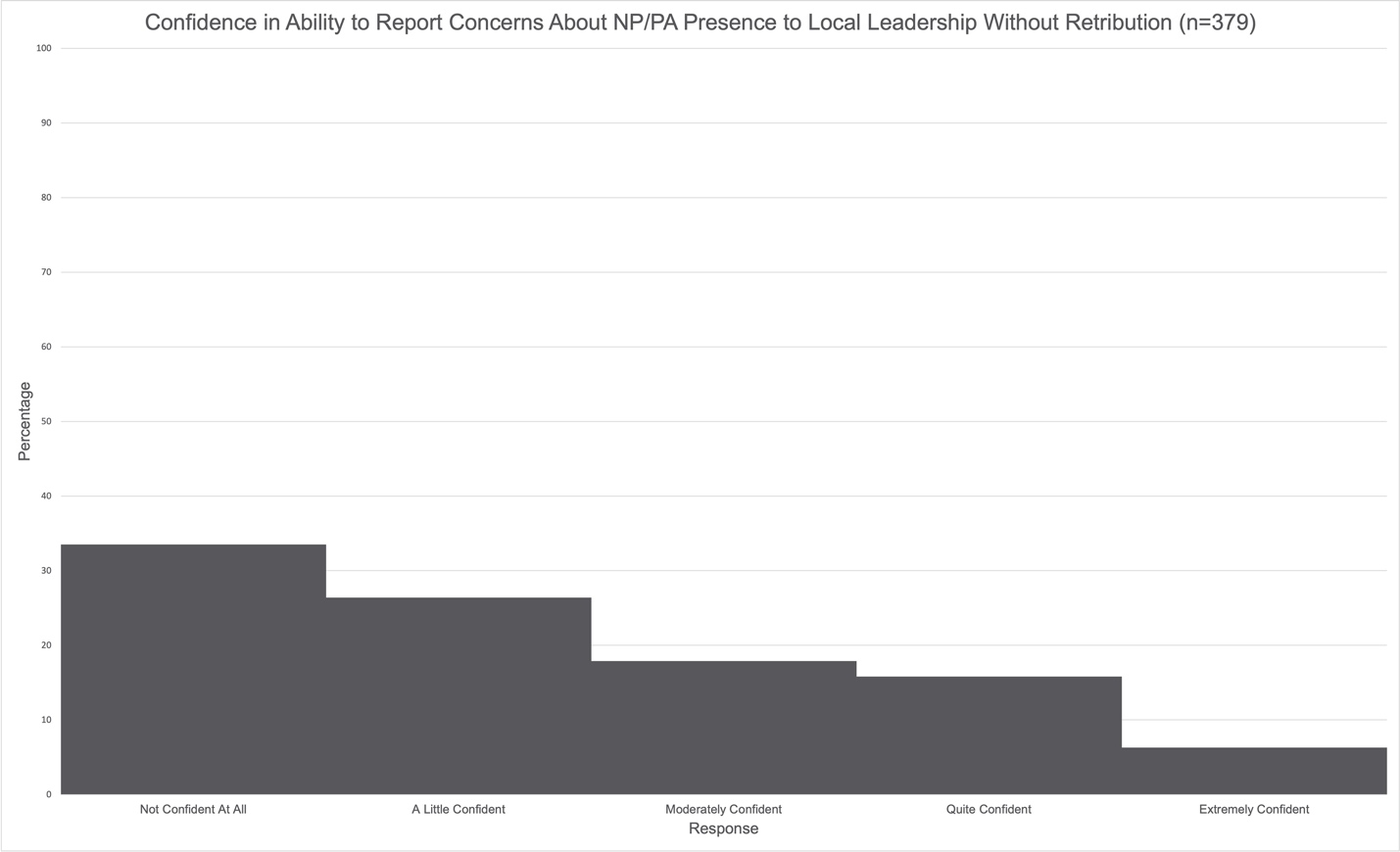

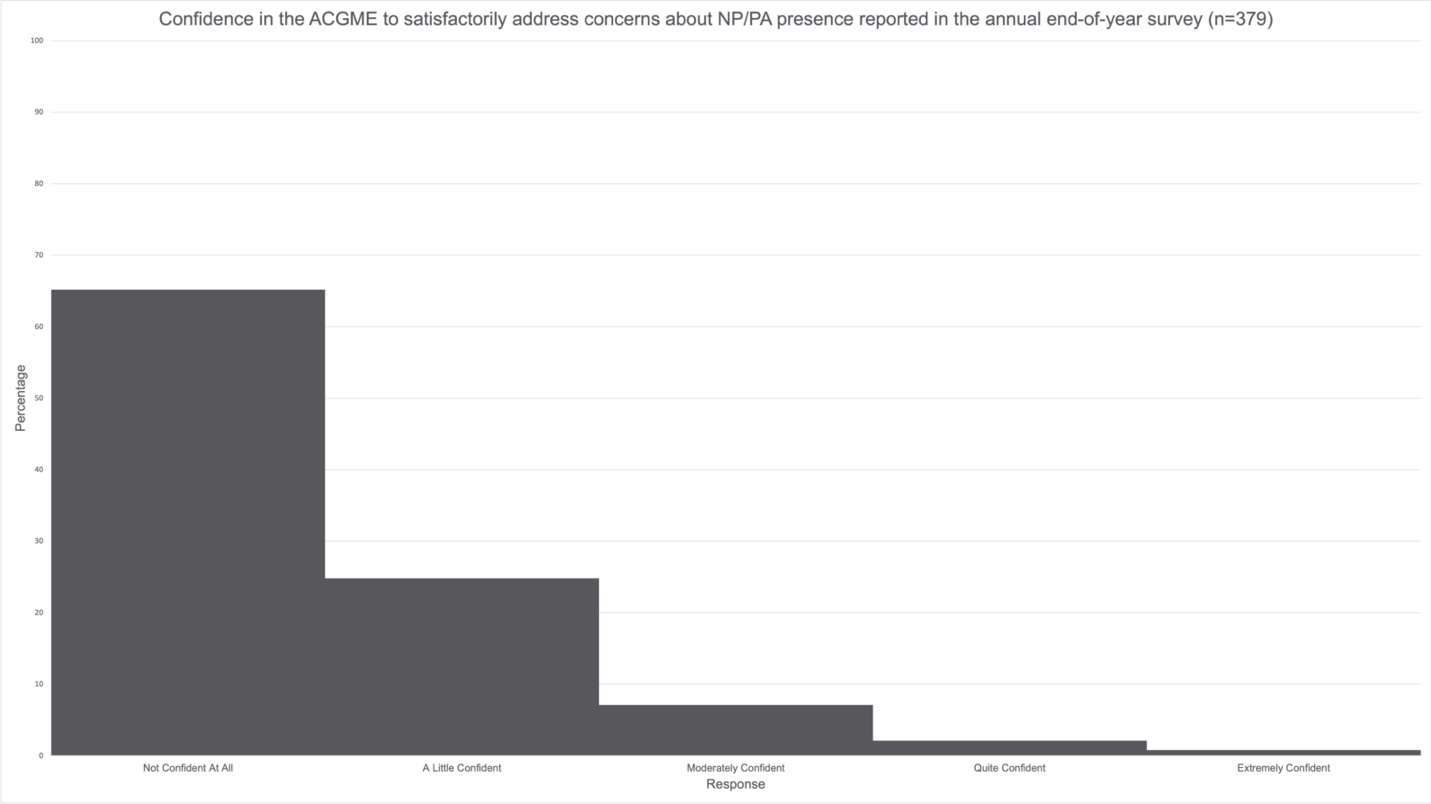

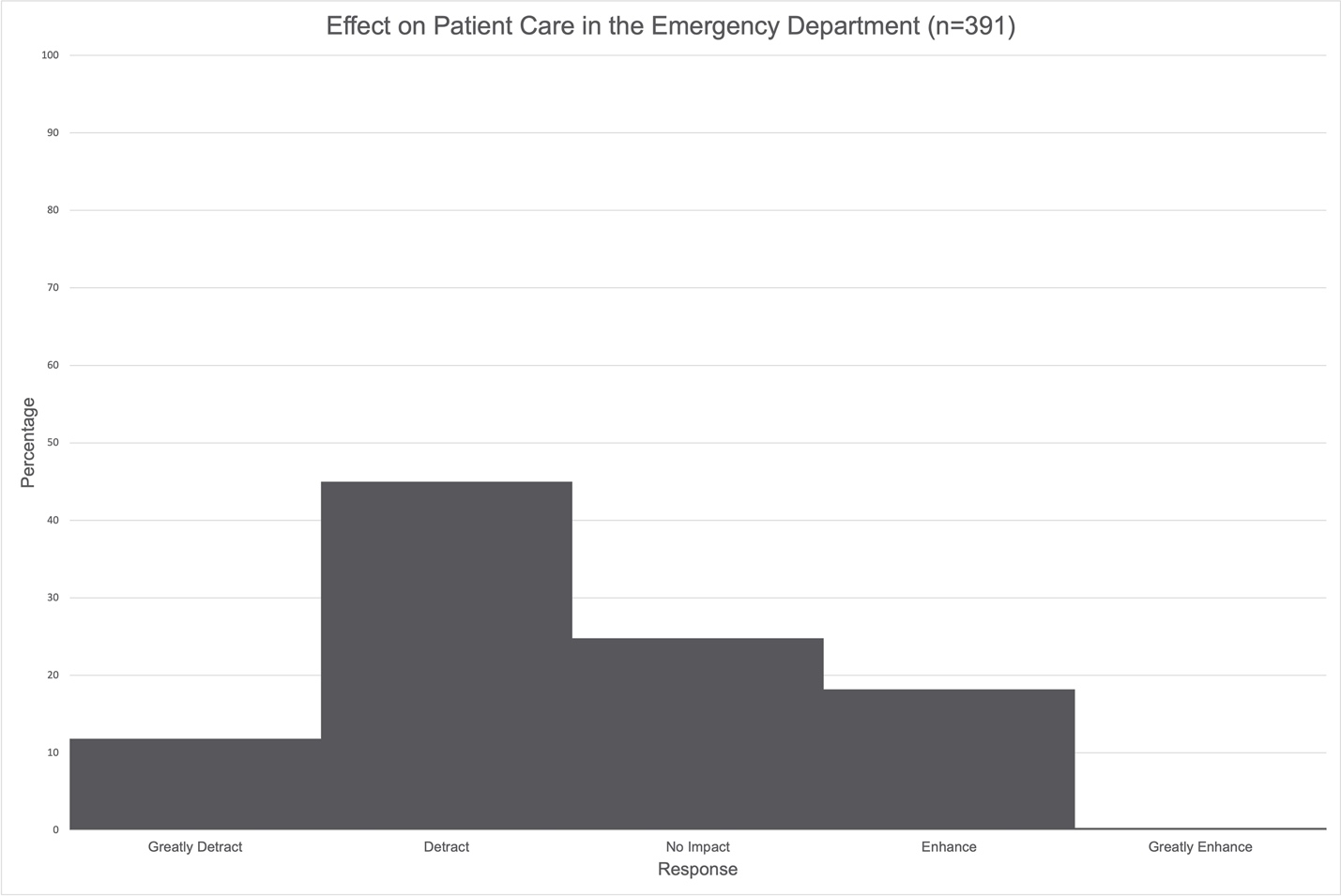

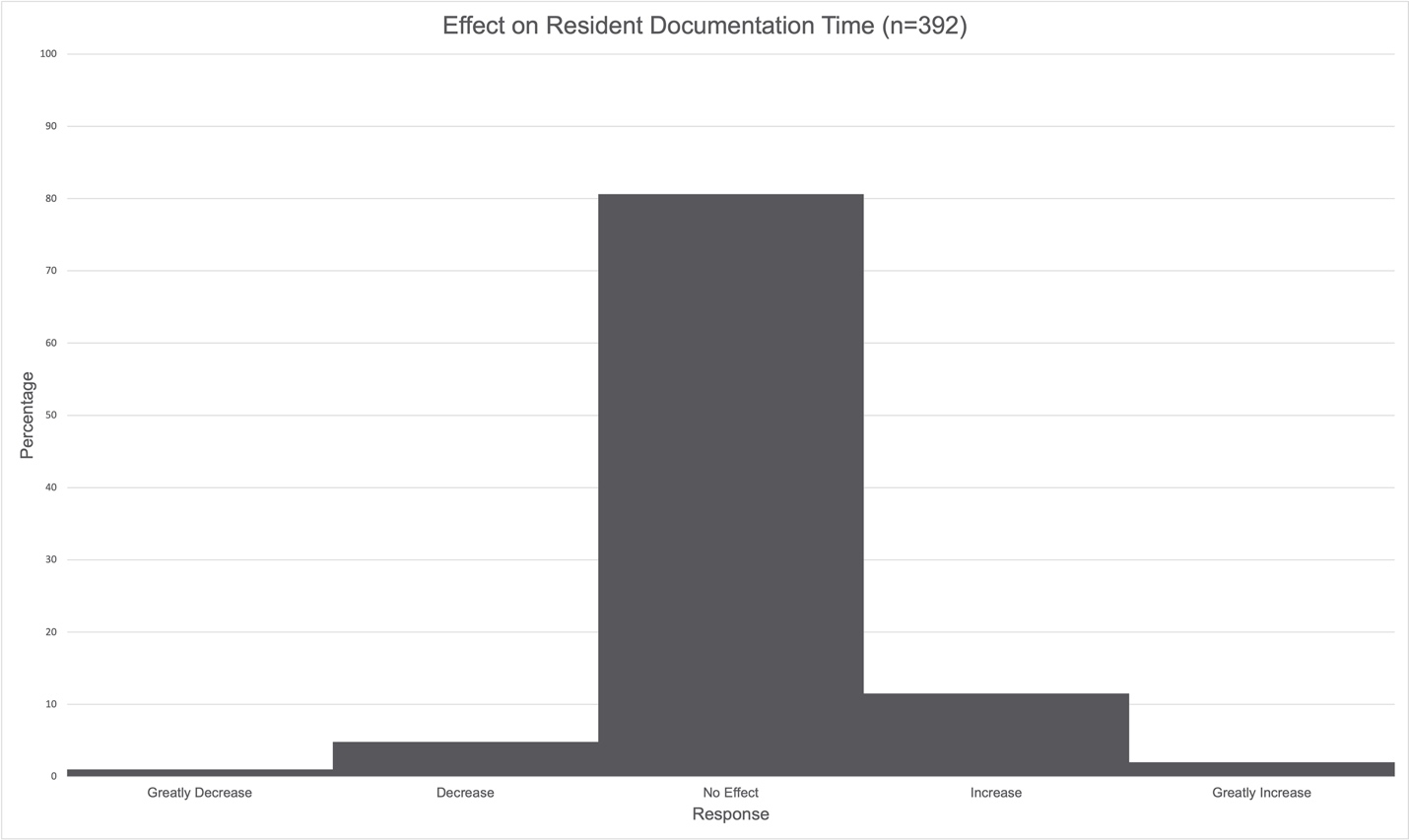

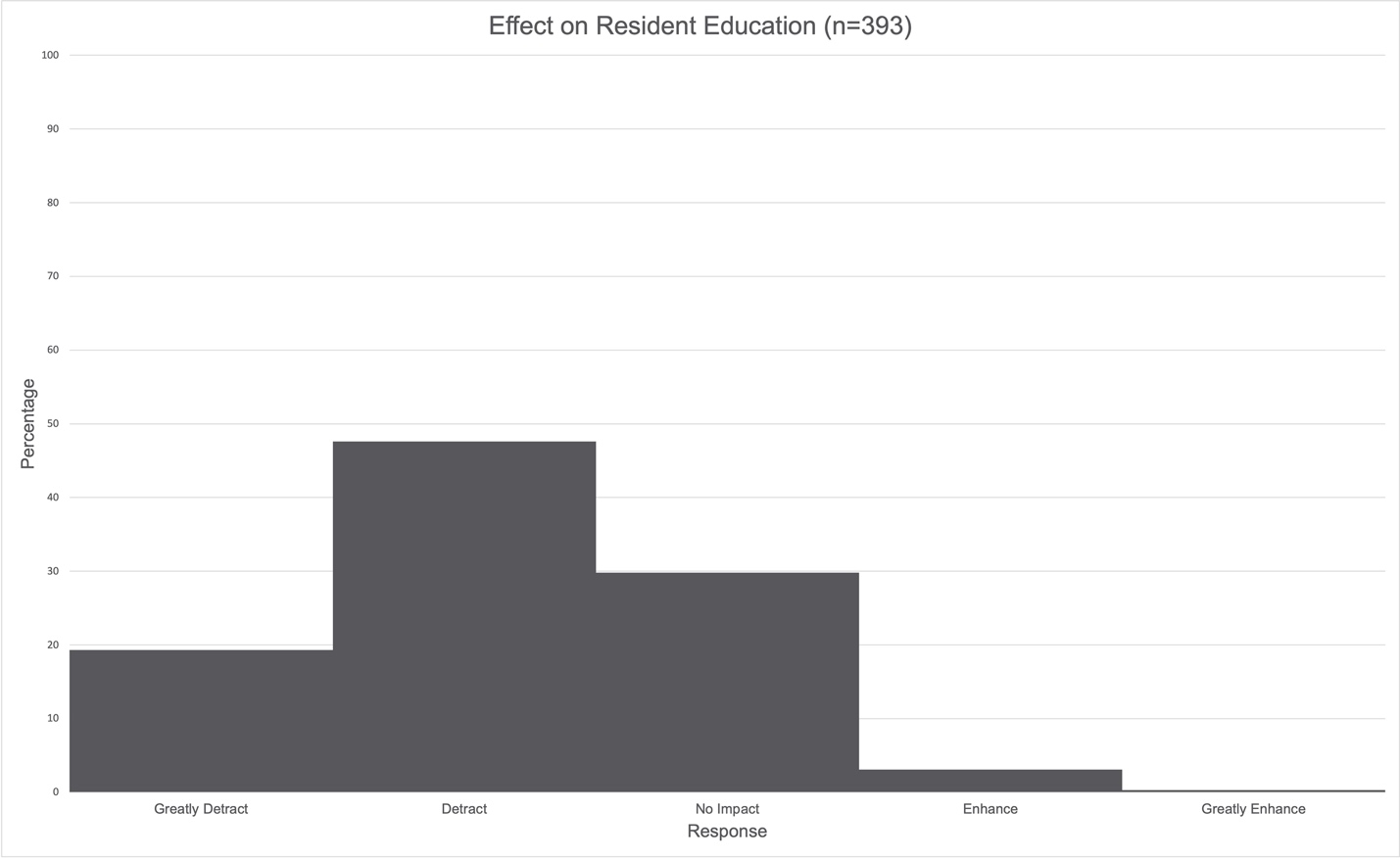

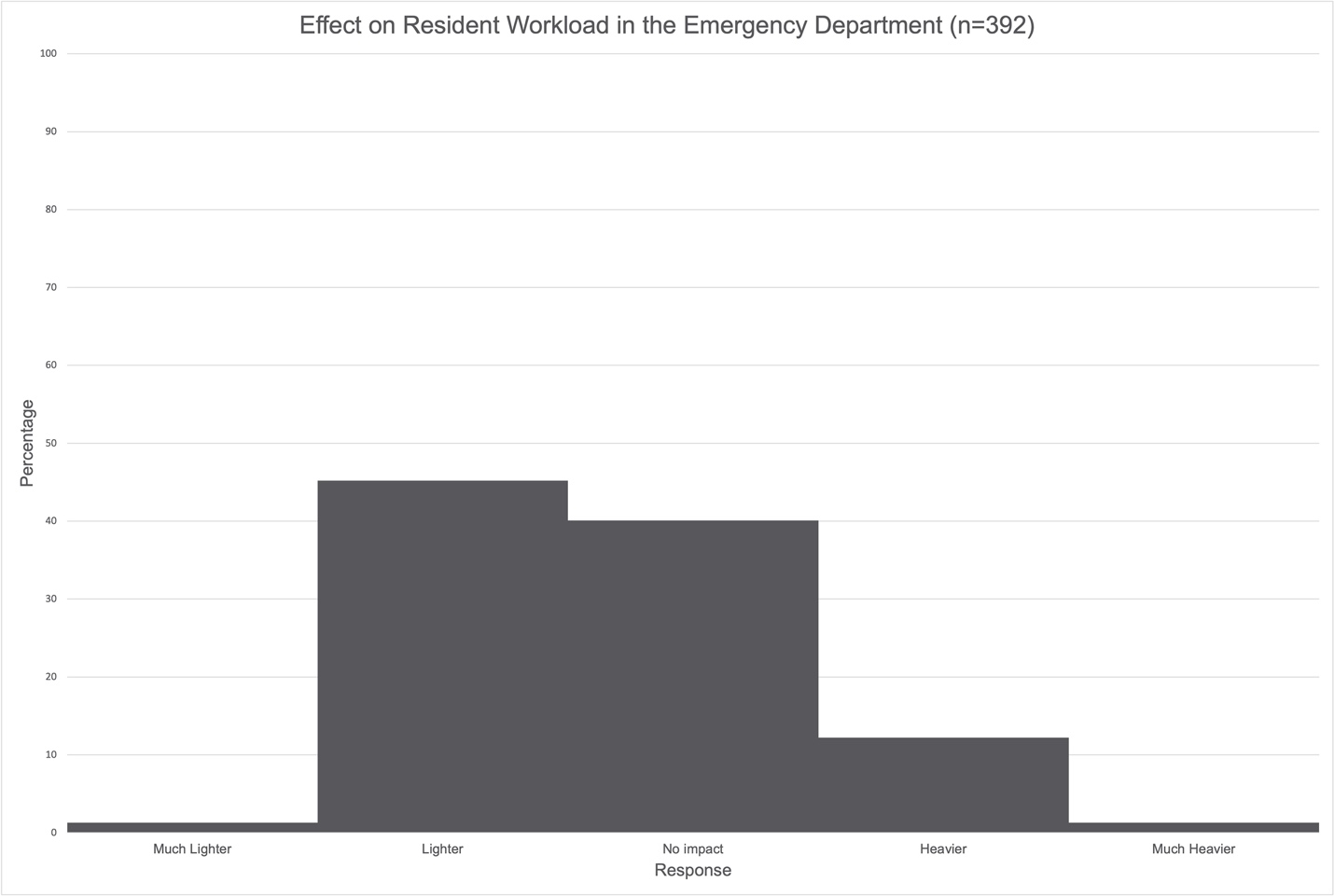

Supplement: Supplementary file 4 [file wjem-24-588-s004.docx]
