# Supplementary material for: Effects of Non-physician Practitioners on Emergency Medicine Physician Resident Education
Source: West J Emerg Med. 2023 May 3;24(3):588–96. doi: 10.5811/westjem.58759 (PMC10284528; doi:10.5811/westjem.58759)

| **Procedure** | **Setting, n** | **Minimum, Maximum** | **Median** | **Mode** | **% Reporting Nonzero** |
| --- | --- | --- | --- | --- | --- |
| Adult medical resuscitation | ED, n=261 | 0,50 | 0.00 | 0 | 21.5% |
|  | OS, n=211 | 0,99 | 0.00 | 0 | 21.3% |
| Adult trauma resuscitation | ED, n=262 | 0,50 | 0.00 | 0 | 23.3% |
|  | OS, n=215 | 0,99 | 0.00 | 0 | 19.1% |
| Cardiac pacing | ED, n=253 | 0,8 | 0.00 | 0 | 6.3% |
|  | OS, n=202 | 0,3 | 0.00 | 0 | 1.5% |
| Central venous access | ED, n=268 | 0,50 | 0.00 | 0 | 27.2% |
|  | OS, n=225 | 0,99 | 0.00 | 0 | 40.4% |
| Chest tubes | ED, n=266 | 0,99 | 0.00 | 0 | 24.8% |
|  | OS, n=216 | 0,99 | 0.00 | 0 | 24.1% |
| Cricothyrotomy | ED, n=251 | 0,1 | 0.00 | 0 | 1.2% |
|  | OS, n=201 | 0,1 | 0.00 | 0 | 0.5% |
| Dislocation reduction | ED, n=262 | 0,40 | 0.00 | 0 | 28.2% |
|  | OS, n=203 | 0,99 | 0.00 | 0 | 8.9% |
| Bedside ultrasound | ED, n=259 | 0,99 | 0.00 | 0 | 25.1% |
|  | OS, n=205 | 0,25 | 0.00 | 0 | 8.8% |
| Intubations | ED, n=267 | 0,50 | 0.00 | 0 | 25.8% |
|  | OS, n=215 | 0,99 | 0.00 | 0 | 29.8% |
| Lumbar puncture | ED, n=257 | 0,50 | 0.00 | 0 | 17.9% |
|  | OS, n=202 | 0,10 | 0.00 | 0 | 6.9% |
| Pediatric medical resuscitation | ED, n=256 | 0,40 | 0.00 | 0 | 14.1% |
|  | OS, n=204 | 0,20 | 0.00 | 0 | 9.3% |
| Pediatric trauma resuscitation | ED, n=254 | 0,50 | 0.00 | 0 | 7.5% |
|  | OS, n=202 | 0,16 | 0.00 | 0 | 6.4% |
| Pericardiocentesis | ED, n=252 | 0,2 | 0.00 | 0 | 1.2% |
|  | OS, n=202 | 0,1 | 0.00 | 0 | 0.5% |
| Procedural sedation | ED, n=256 | 0,30 | 0.00 | 0 | 11.7% |
|  | OS, n=203 | 0,20 | 0.00 | 0 | 3.9% |
| Vaginal delivery | ED, n=253 | 0,20 | 0.00 | 0 | 4.7% |
|  | OS, n=205 | 0,30 | 0.00 | 0 | 10.2% |

Table 4. Procedures performed by NPs or PAs on patients managed by EM resident physicians in the ED and off-service settings.

ED, emergency department; OS, off-service


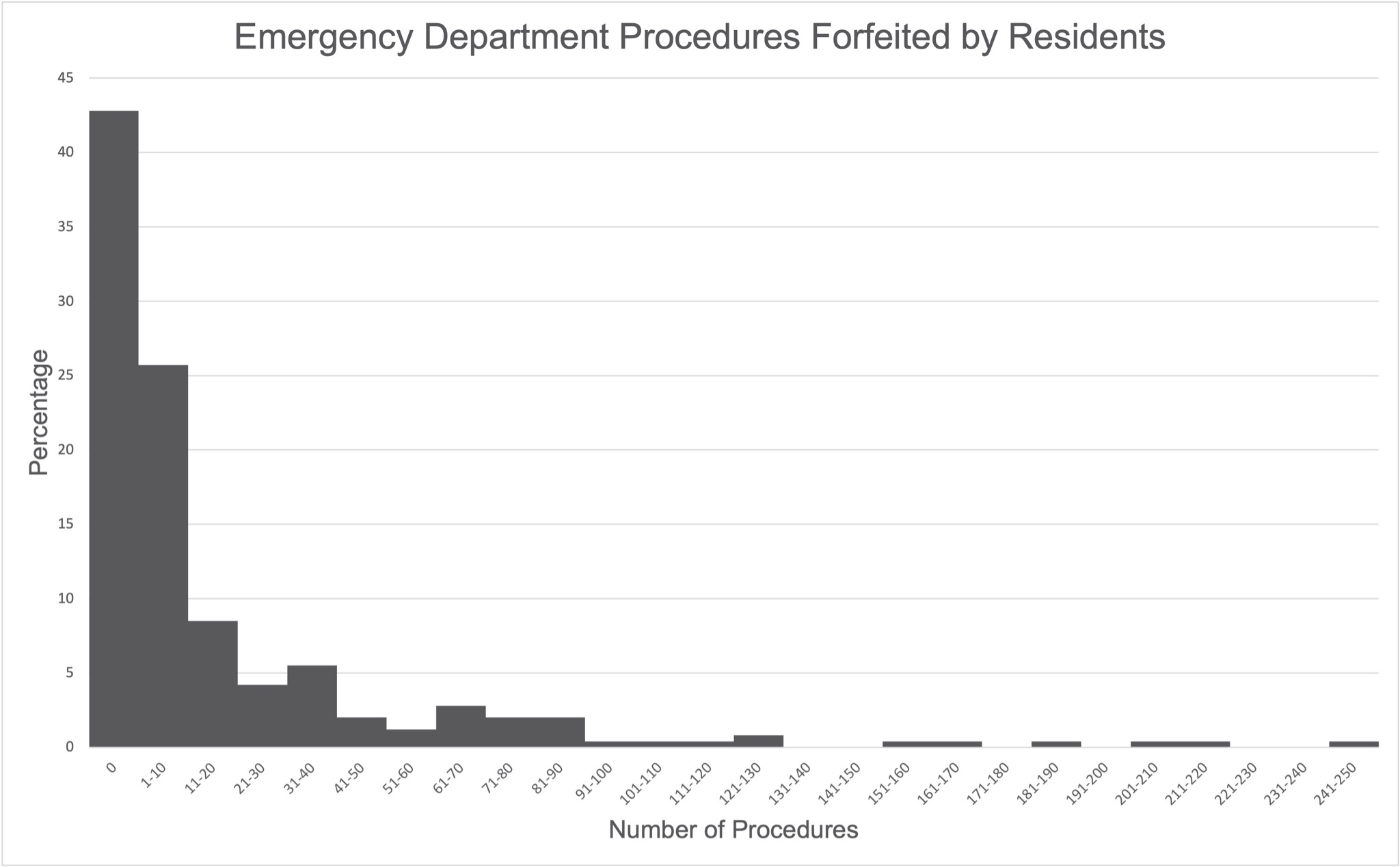

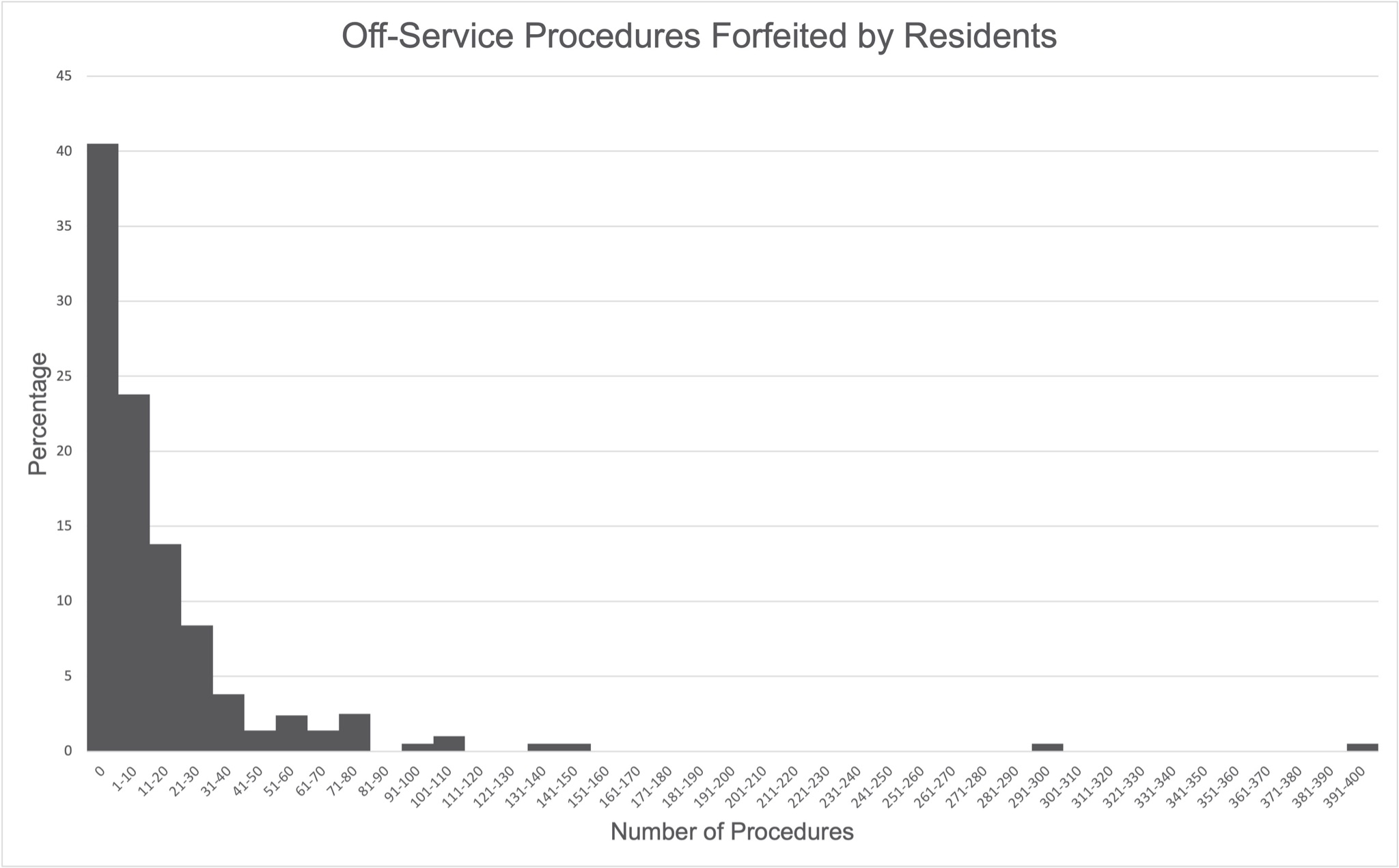


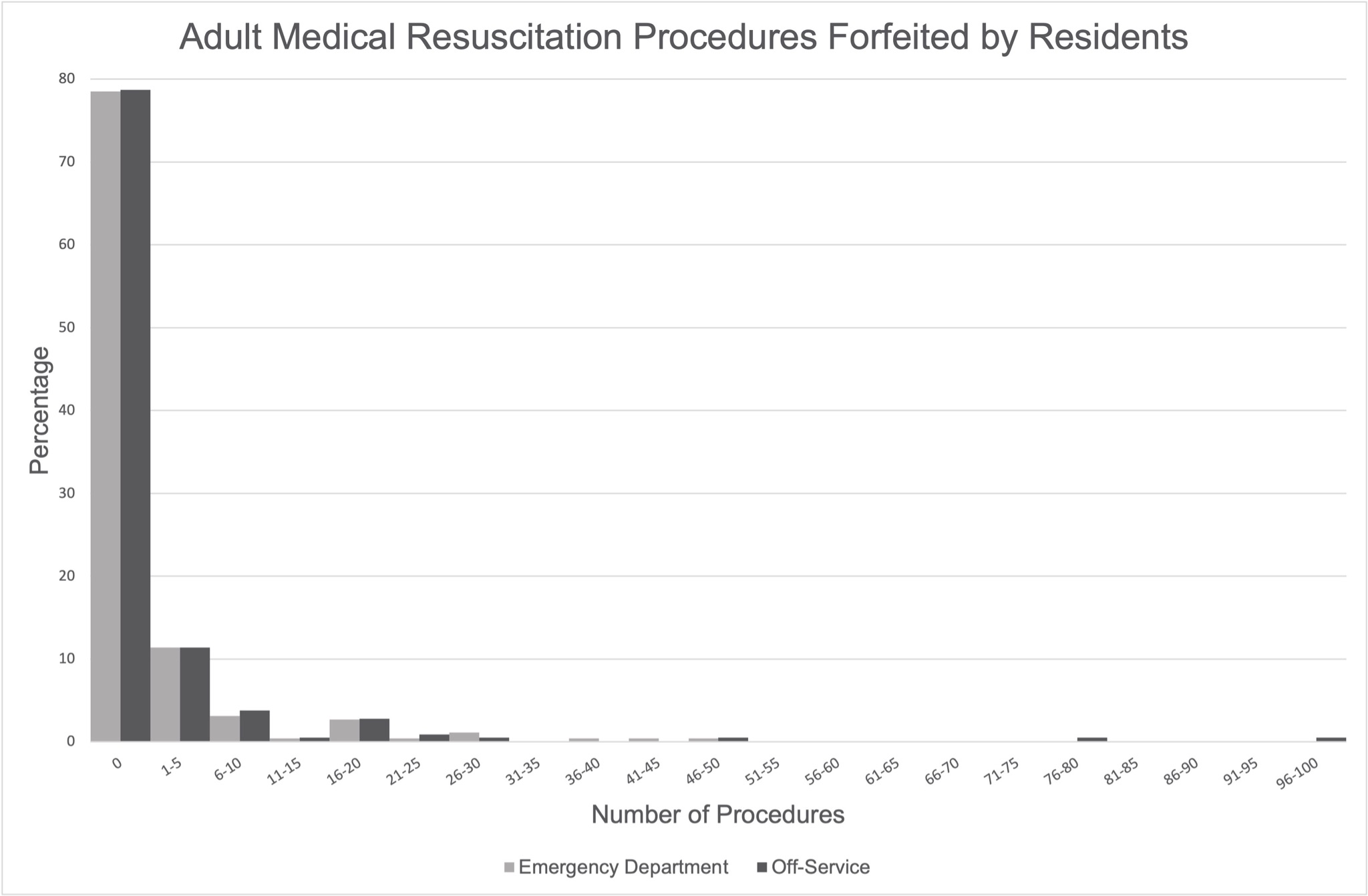

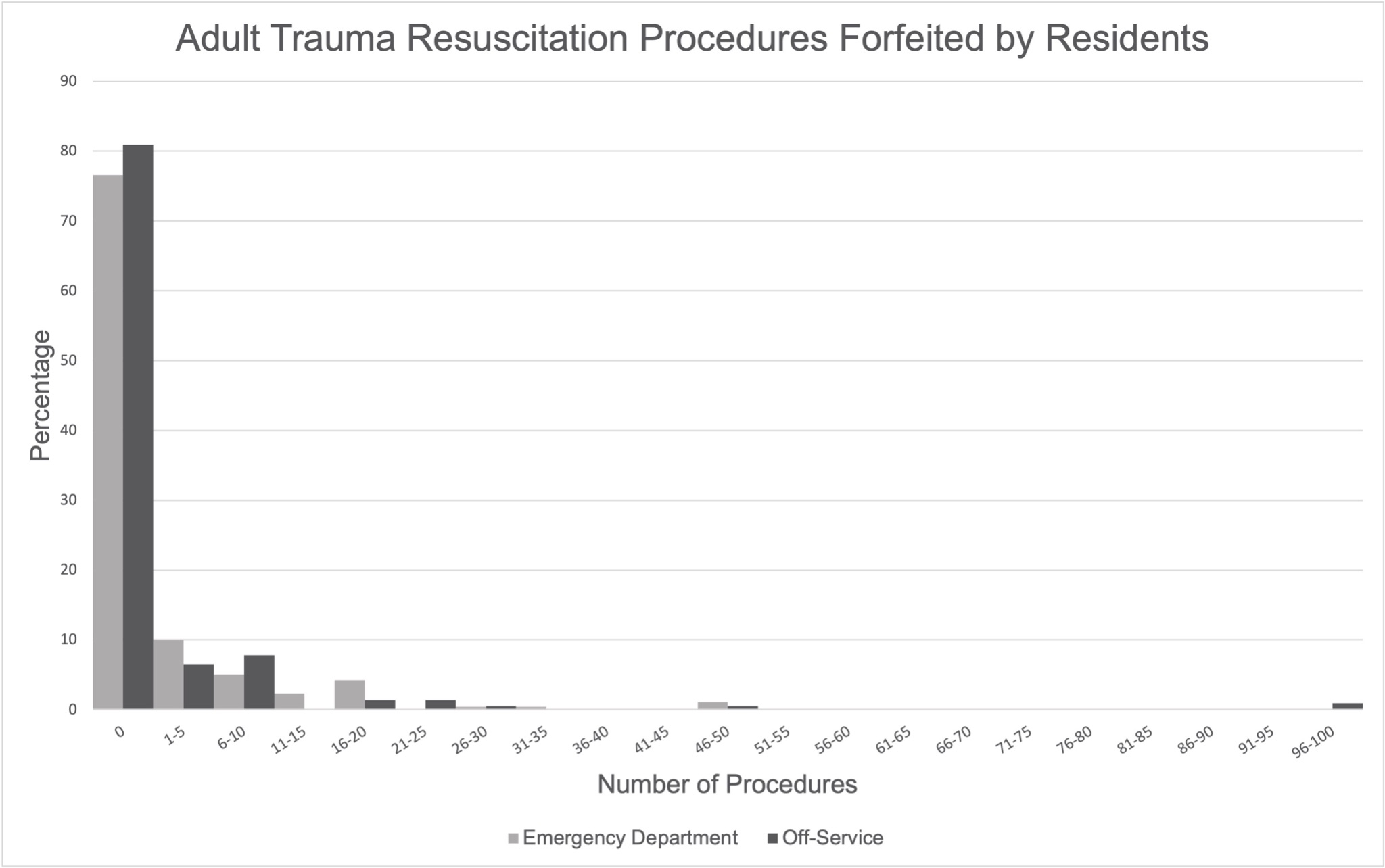

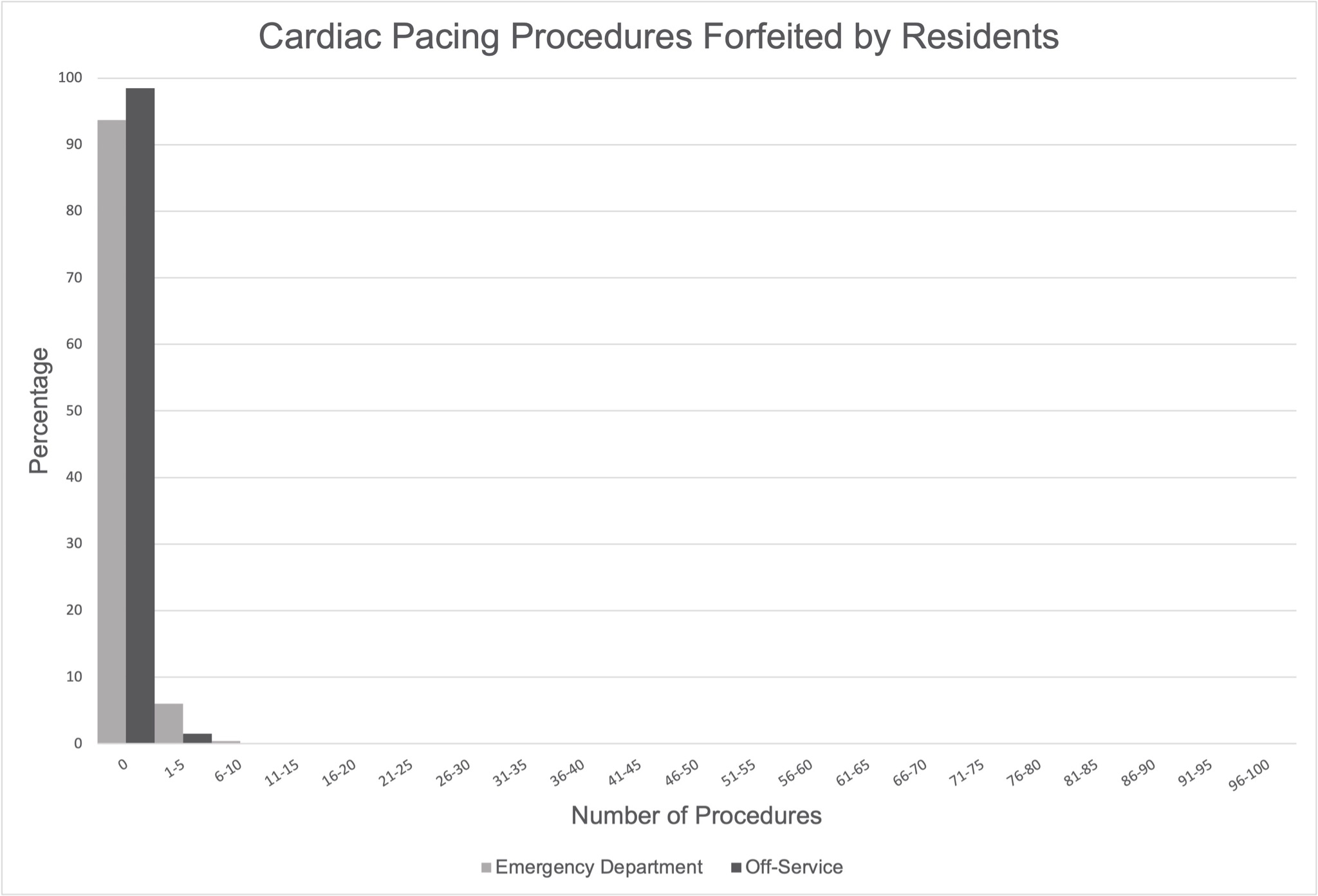

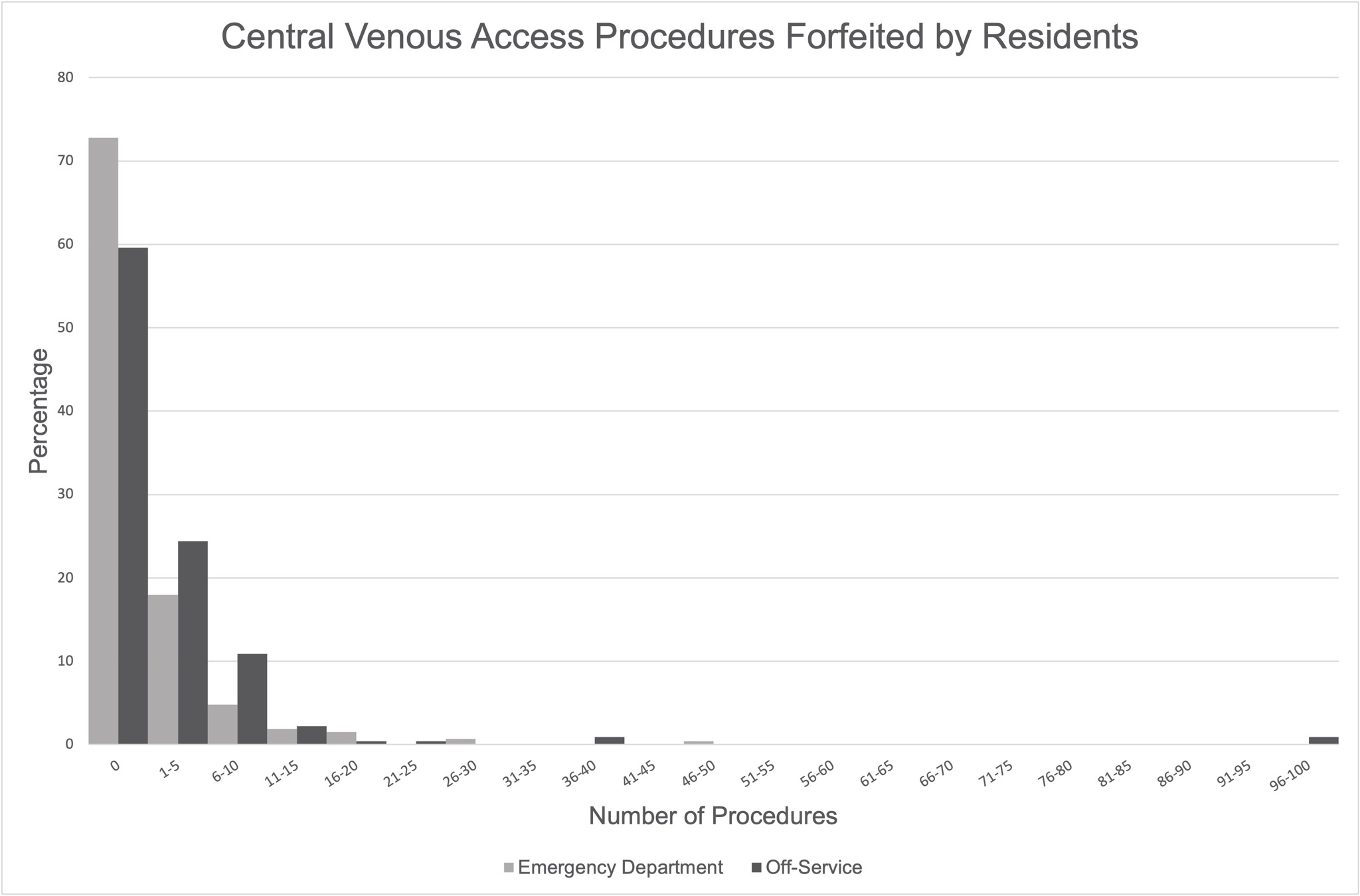

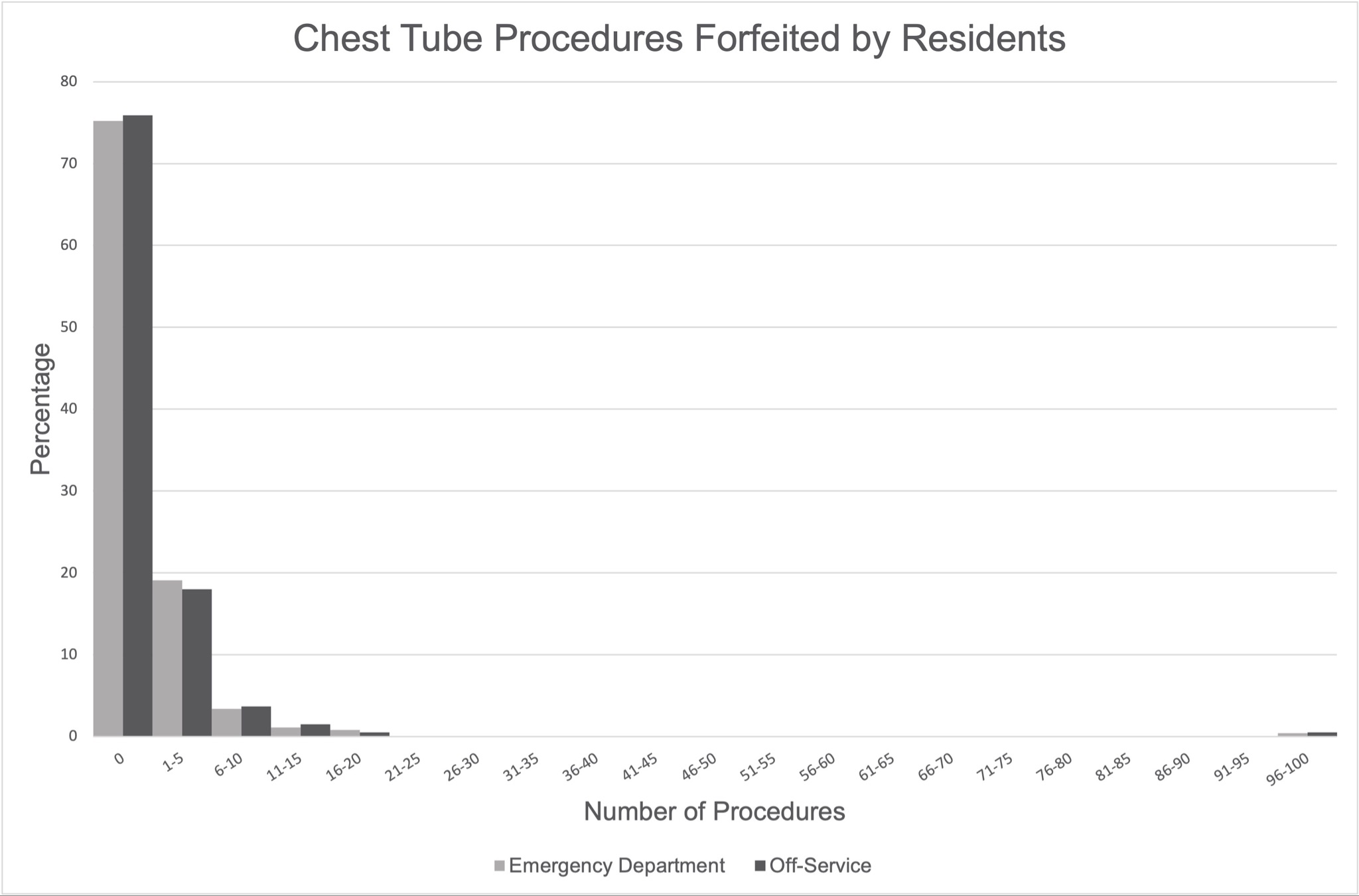

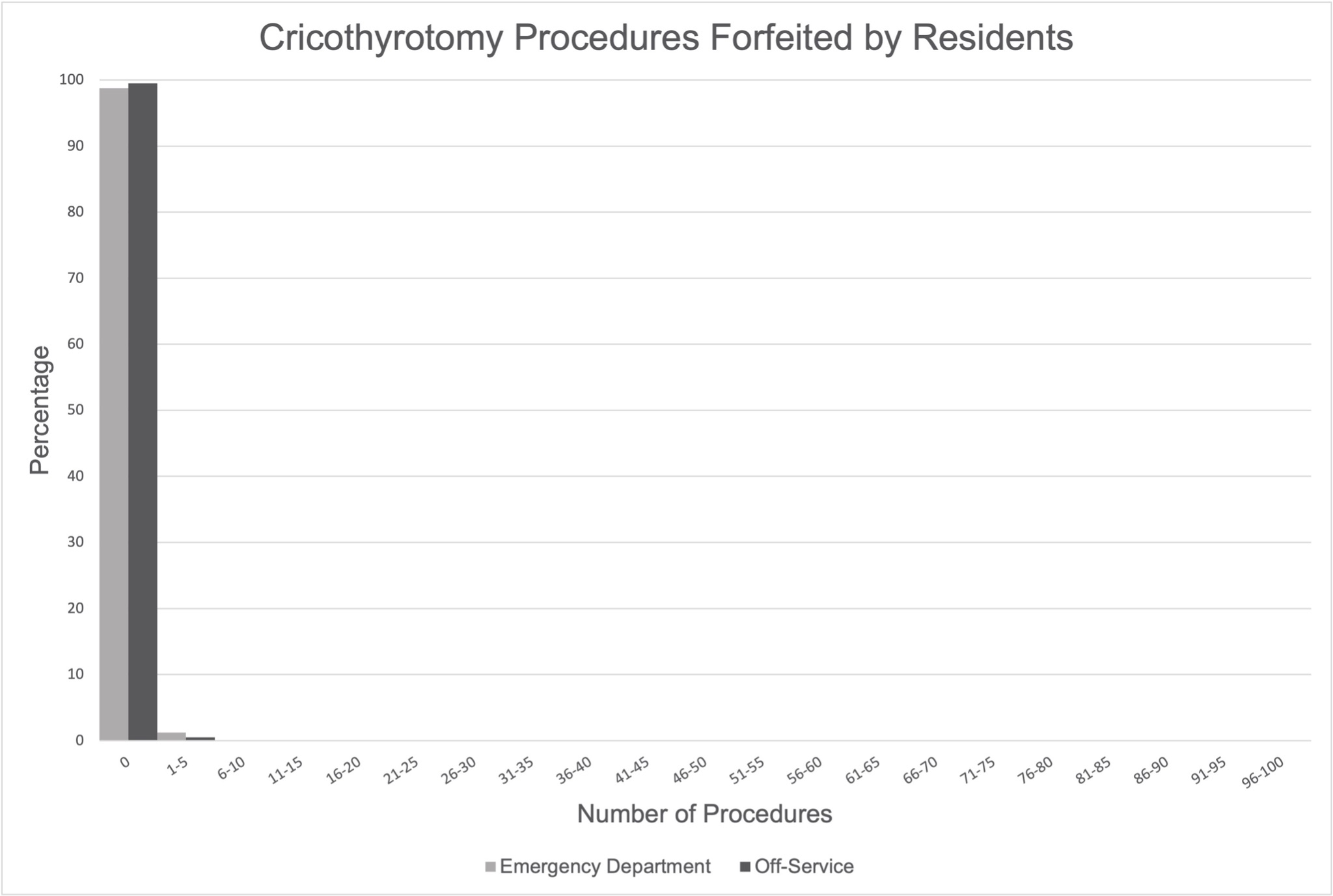

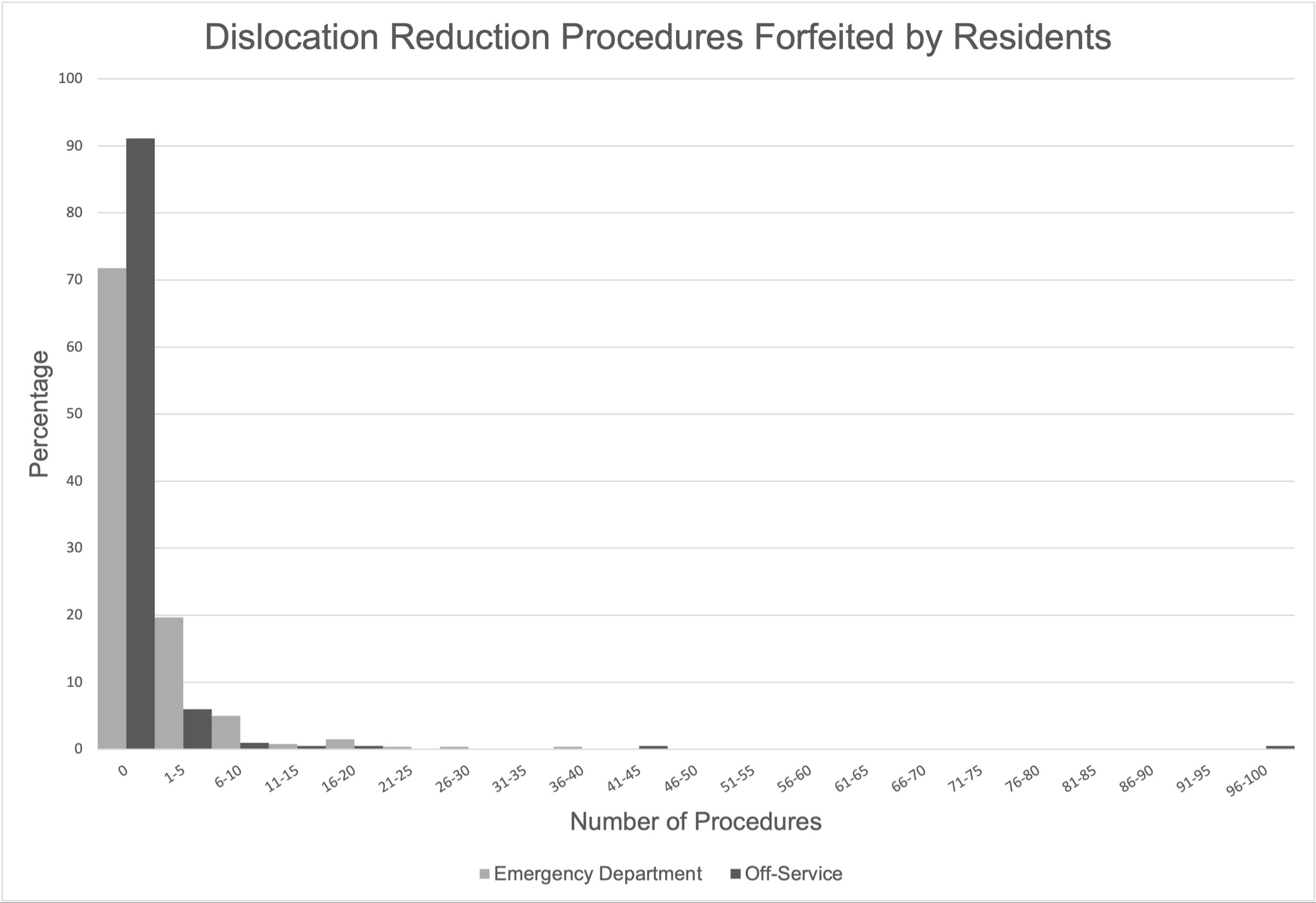

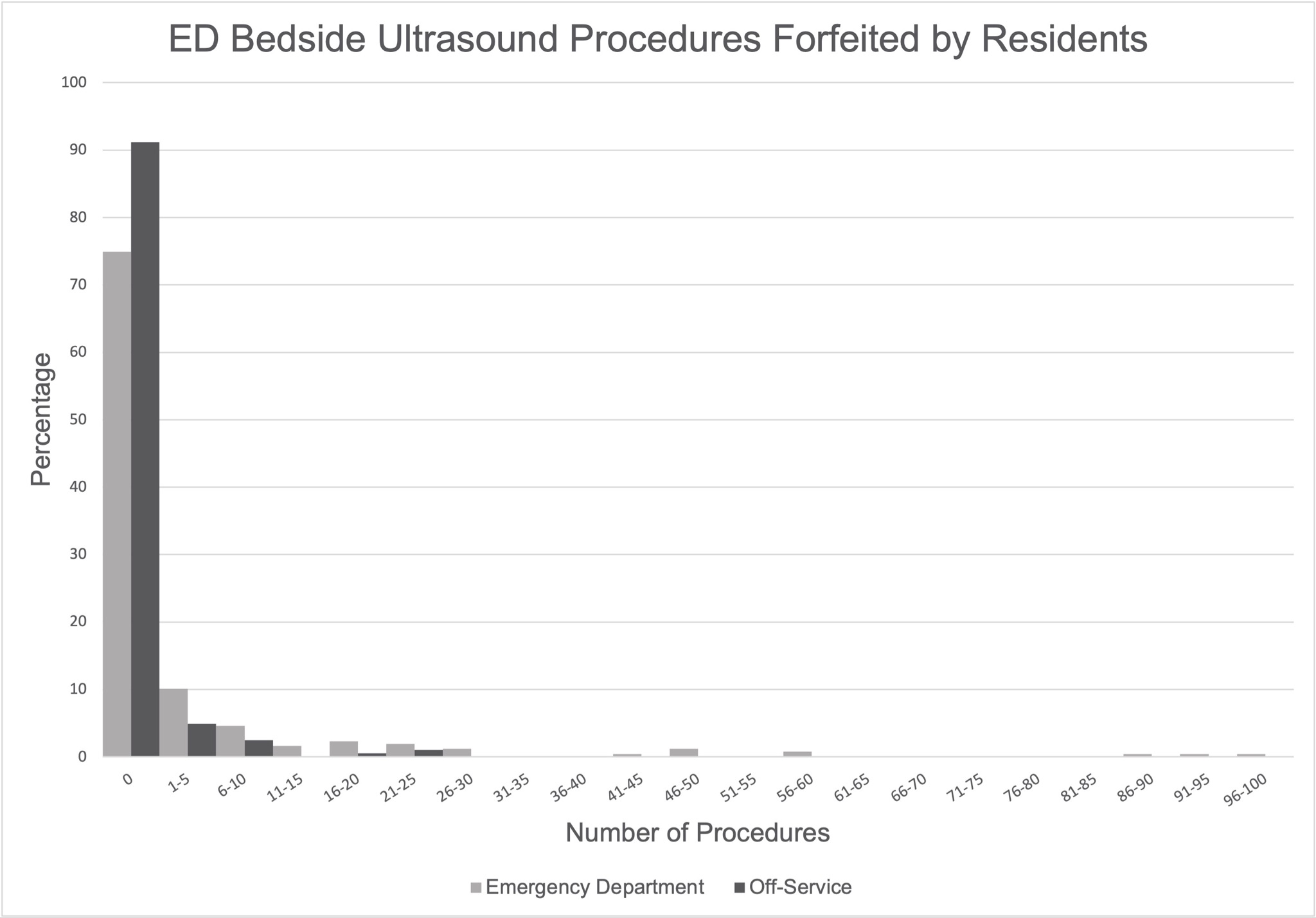

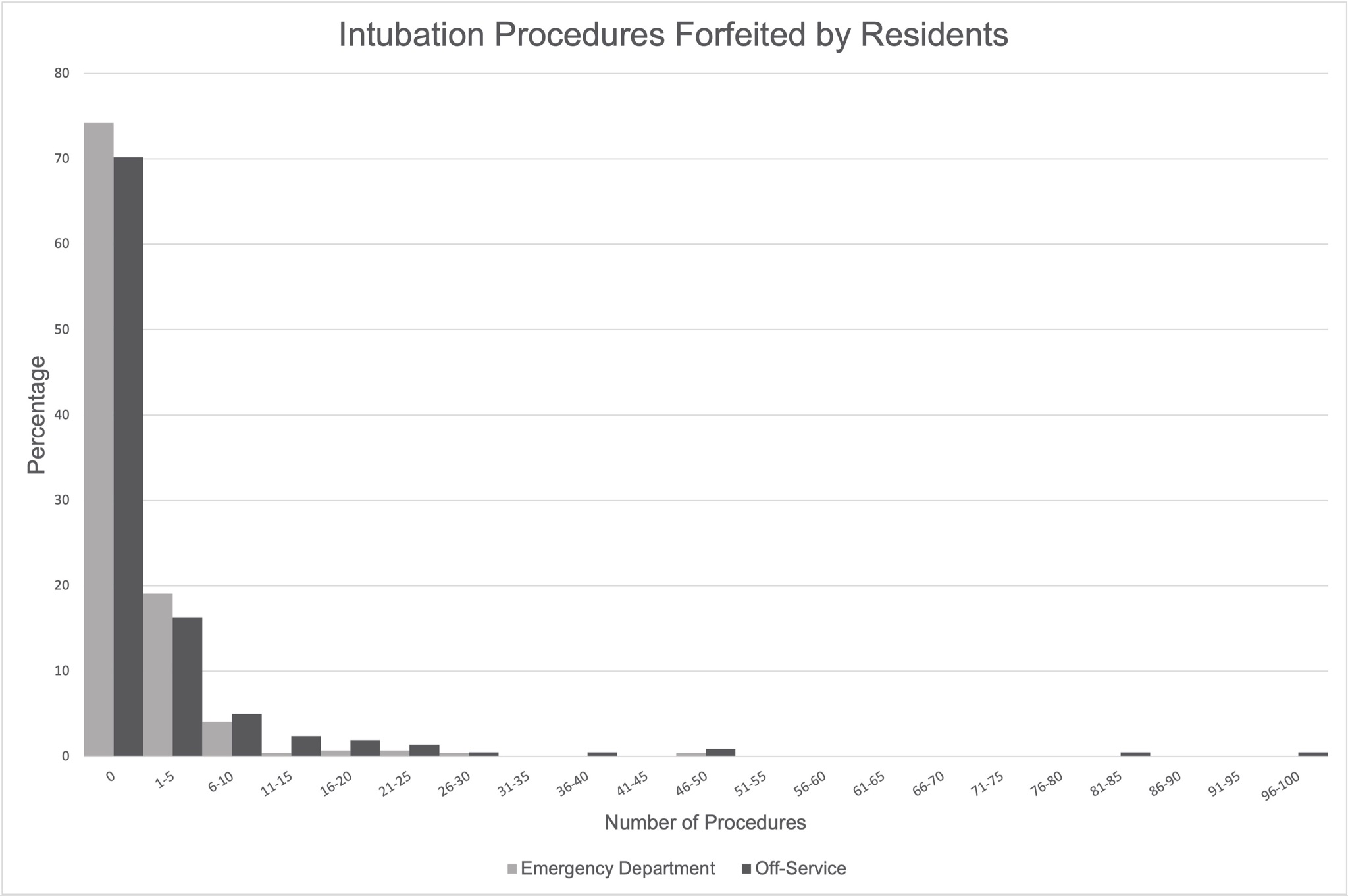

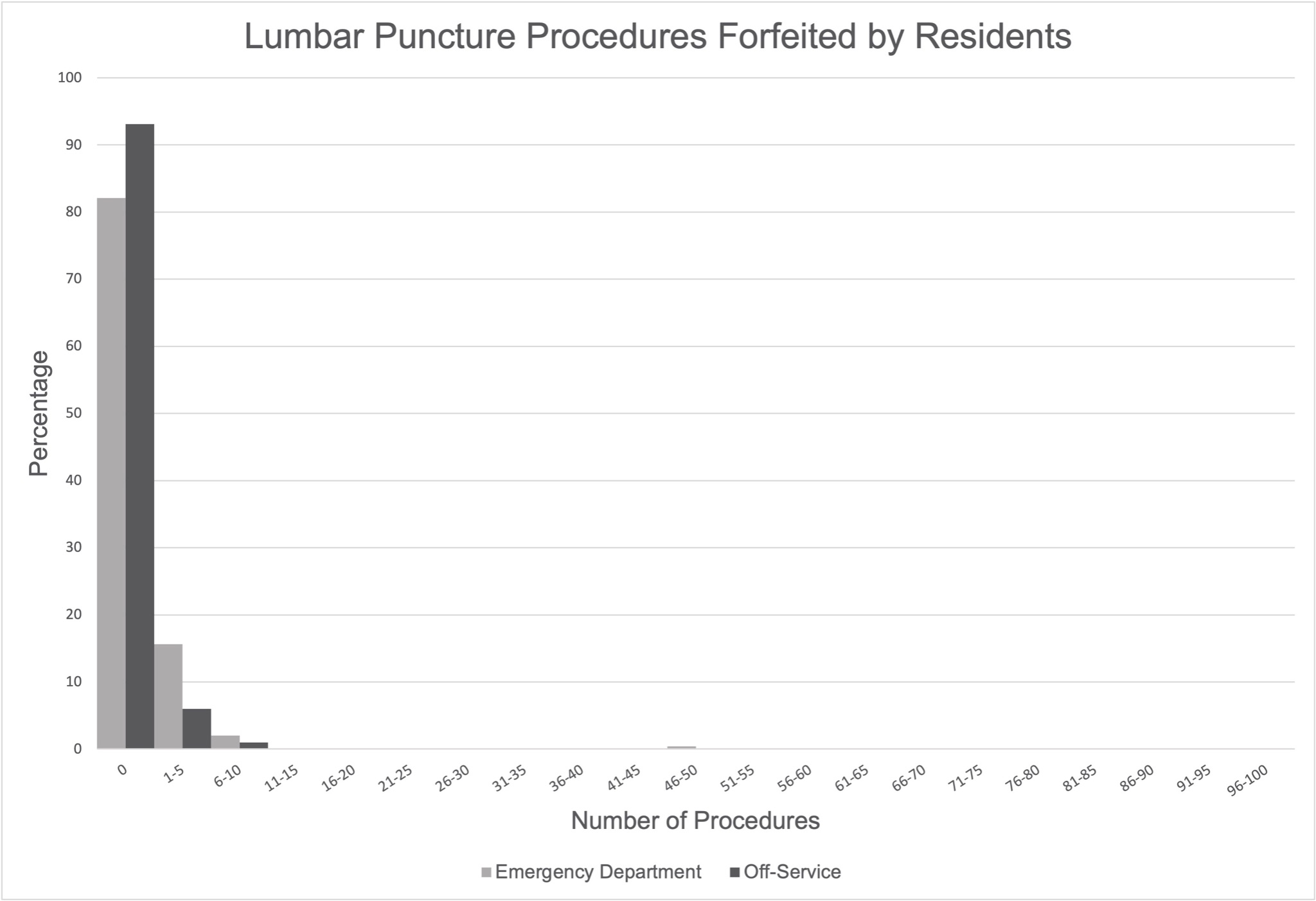

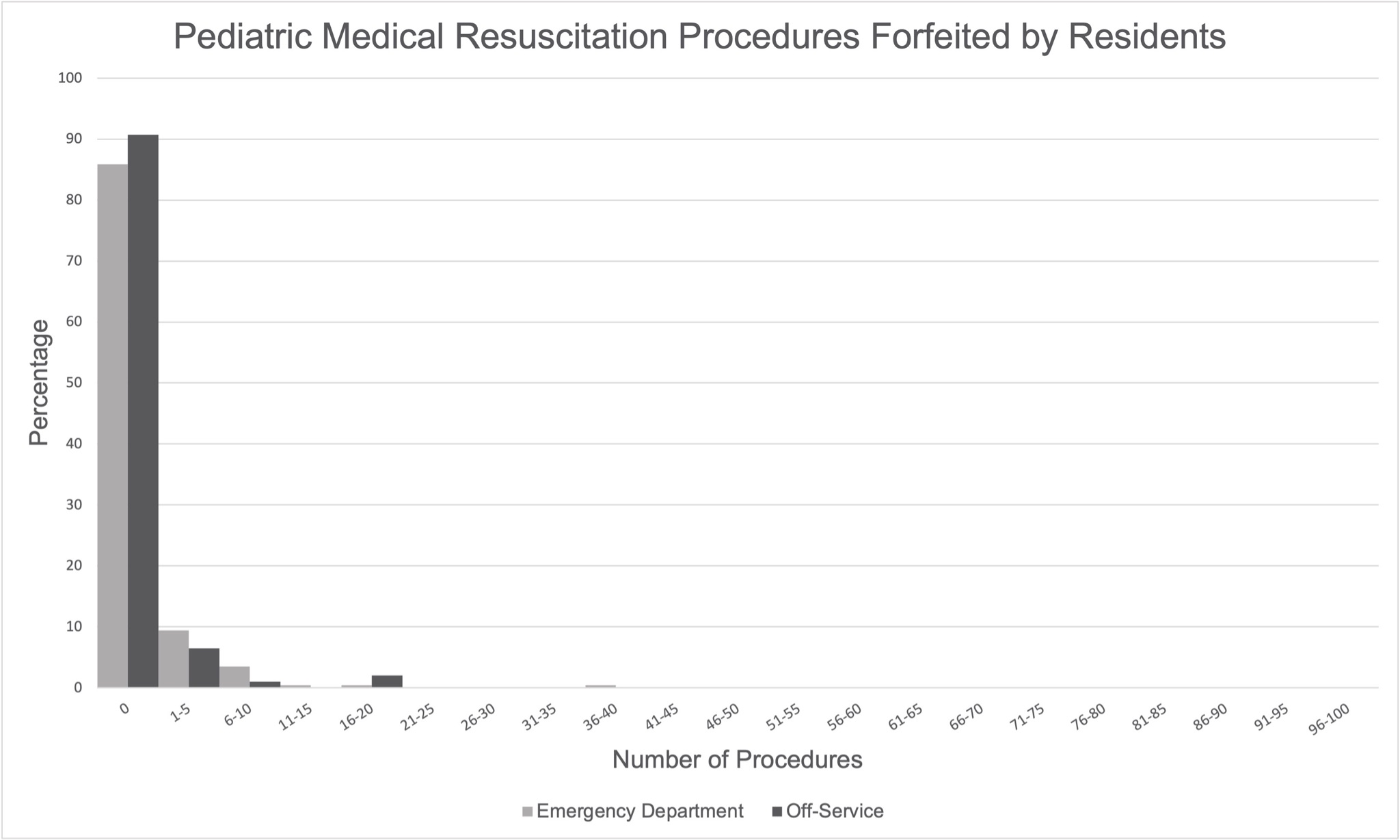

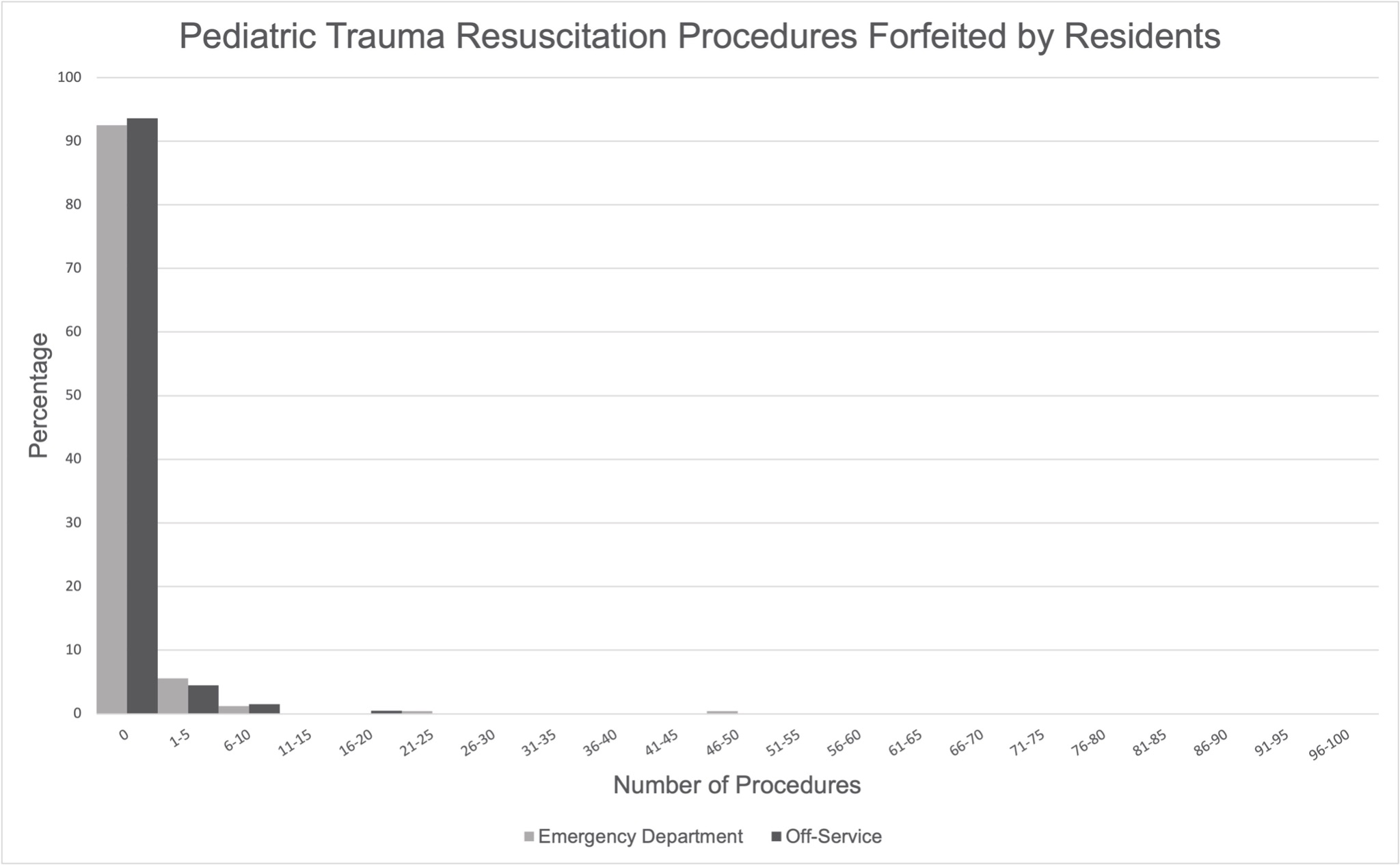

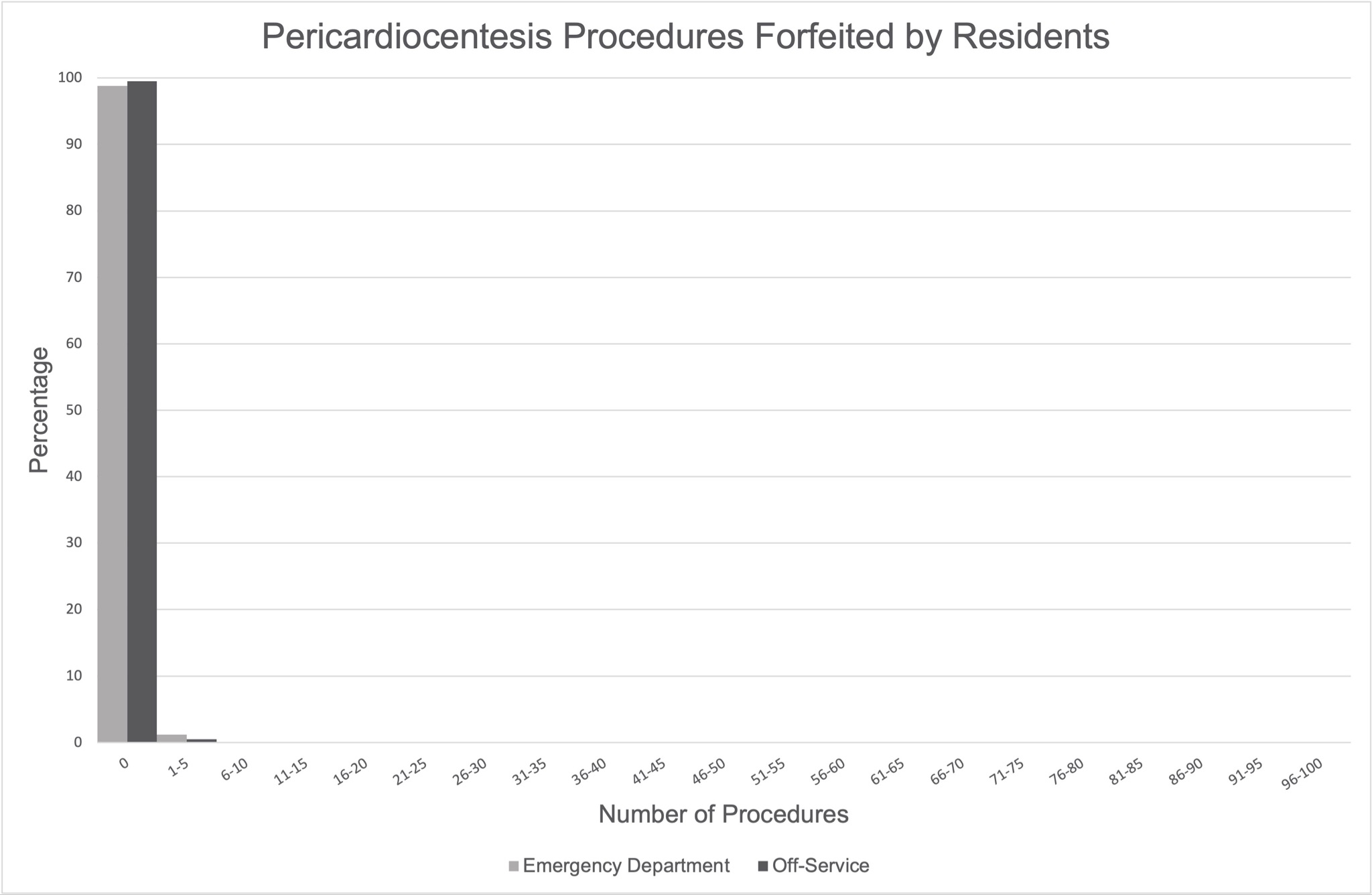

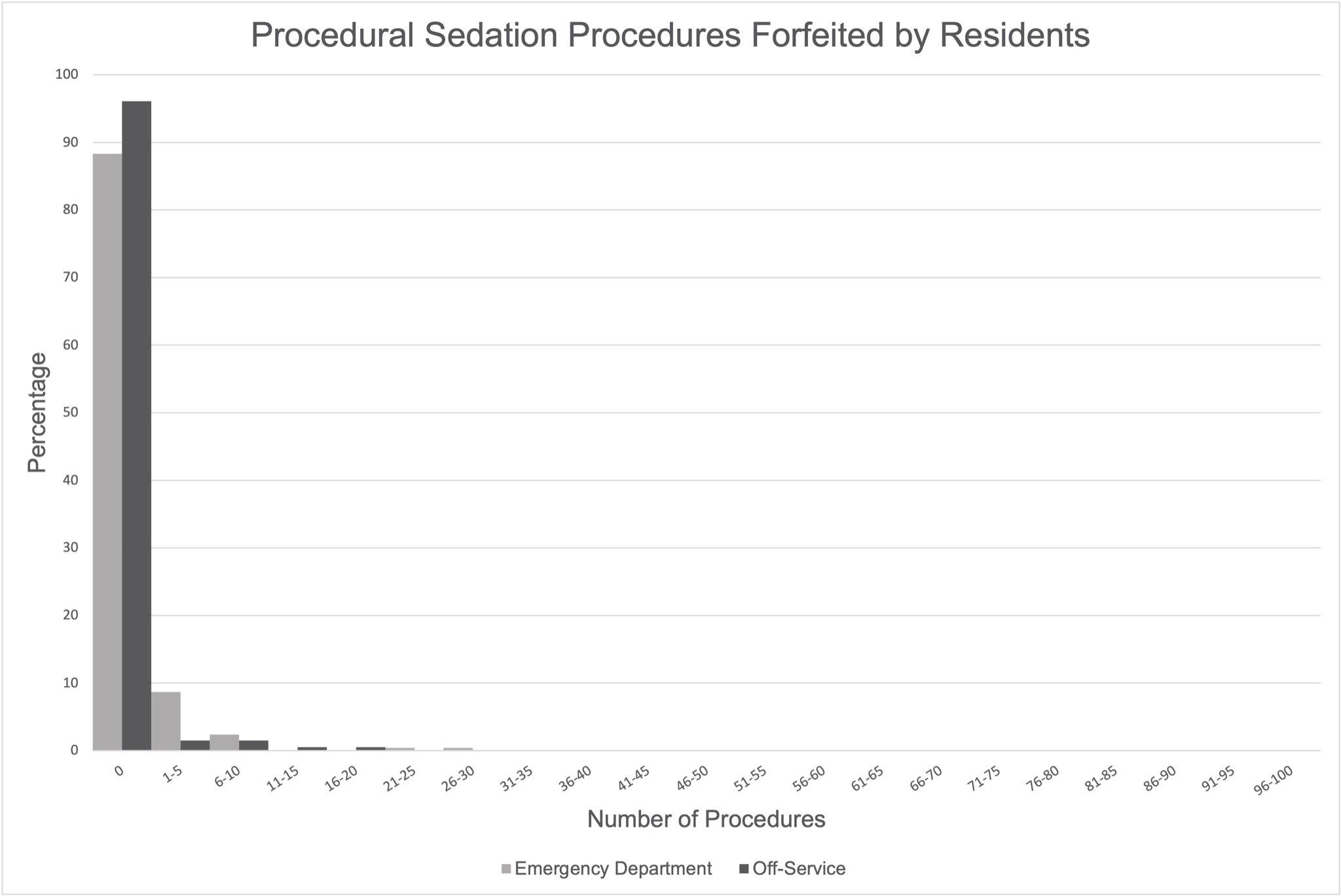

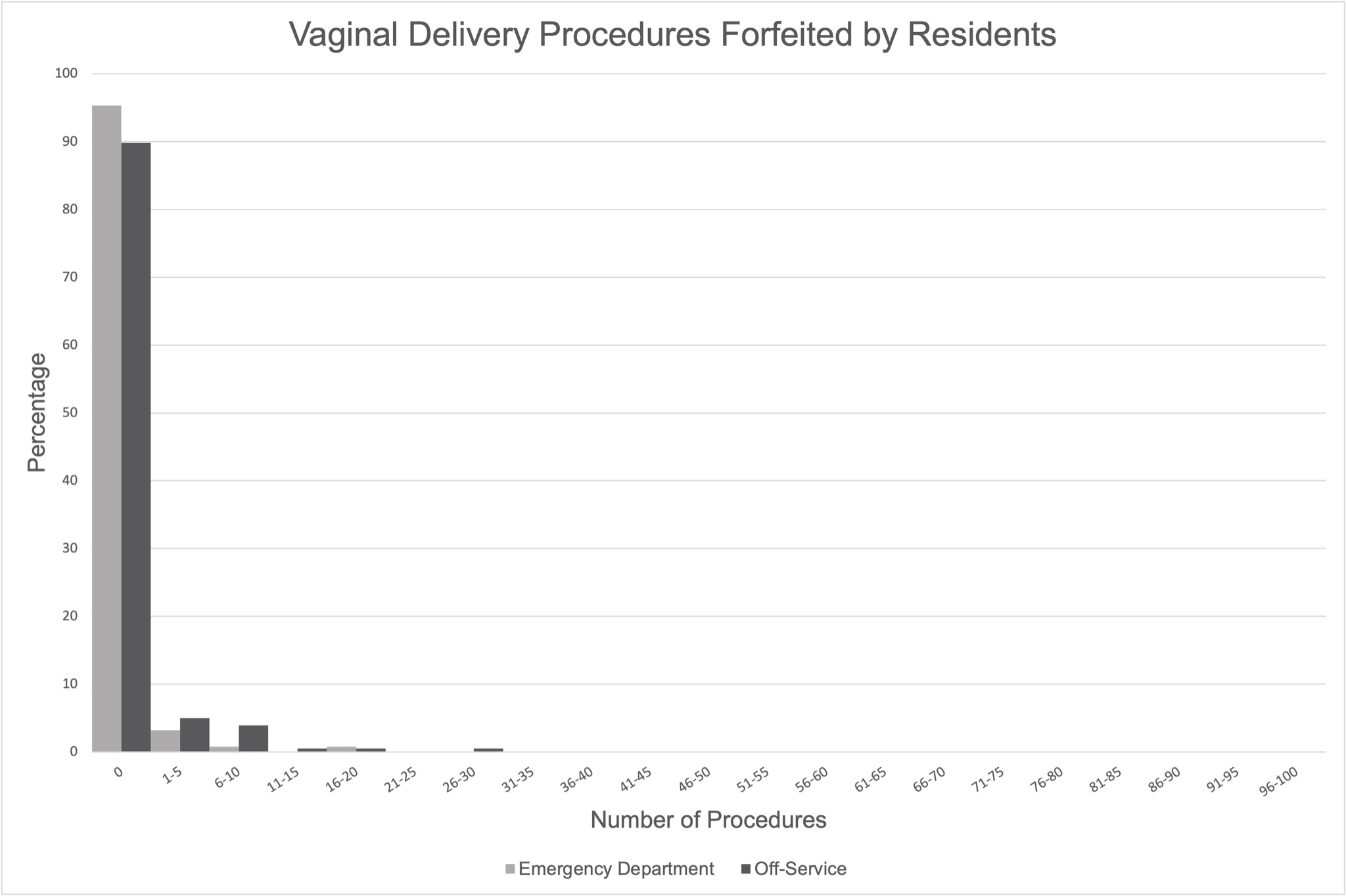

Supplement: Supplementary file 5 [file wjem-24-588-s005.docx]
